# MiR319 mediated salt tolerance by ethylene

**DOI:** 10.1111/pbi.13154

**Published:** 2019-06-07

**Authors:** Yanrong Liu, Dayong Li, Jianping Yan, Kexin Wang, Hong Luo, Wanjun Zhang

**Affiliations:** ^1^ Department of Grassland Science China Agricultural University Beijing China; ^2^ Beijing Vegetable Research Center (BVRC) Beijing Academy of Agricultural and Forestry Sciences National Engineering Research Center for Vegetables Beijing China; ^3^ Department of Genetics and Biochemistry Clemson University Clemson SC USA; ^4^ National Energy R&D Center for Biomass (NECB) China Agricultural University Beijing China

**Keywords:** salt tolerance, ethylene, Met cycle, miR319, switchgrass

## Abstract

Salinity‐induced accumulation of certain microRNAs accompanied by gaseous phytohormone ethylene production has been recognized as a mechanism of plant salt tolerance. MicroRNA319 (miR319) has been characterized as an important player in abiotic stress resistance in some C3 plants, such as *Arabidopsis thaliana* and rice. However, its role in the dedicated biomass plant switchgrass (*Panicum virgatum* L.), a C4 plant, has not been reported. Here, we show crosstalk between miR319 and ethylene (ET) for increasing salt tolerance. By overexpressing *Osa‐MIR319b* and a target mimicry form of miR319 (*MIM319*), we showed that miR319 positively regulated ET synthesis and salt tolerance in switchgrass. By experimental treatments, we demonstrated that ET‐mediated salt tolerance in switchgrass was dose‐dependent, and miR319 regulated the switchgrass salt response by fine‐tuning ET synthesis. Further experiments showed that the repression of a miR319 target, *PvPCF5,* in switchgrass also led to enhanced ethylene accumulation and salt tolerance in transgenic plants. Genome‐wide transcriptome analysis demonstrated that overexpression of miR319 (OE‐miR319) down‐regulated the expression of key genes in the methionine (Met) cycle but promoted the expression of genes in ethylene synthesis. The results enrich our understanding of the synergistic effects of the miR319‐*PvPCF5* module and ethylene synthesis in the salt tolerance of switchgrass, a C4 bioenergy plant.

## Introduction

Switchgrass, a warm‐season perennial C4 grass, has been extensively used as a forage crop and a dedicated biomass feedstock plant for bioenergy production in the United States (McLaughlin and Kszos, 2005). However, to avoid competition with crops in land use, it is preferable to grow switchgrass in salinity‐affected or water‐limited marginal non‐agricultural land (Schmer *et al*., 2011). Previous studies showed that the growth and development of switchgrass were restrained significantly by salt stress (Kim *et al*., 2016), under which switchgrass exhibited decreased biomass (Zhuo *et al*., 2015), low germination rate (Wang *et al*., 2012) and a series of physiological reactions in cells (Wang *et al*., 2012; Xie *et al*., 2014). Therefore, improving salt tolerance to utilize marginal land is of primary concern in switchgrass breeding. Recently, although there have been a few papers reporting improved salt tolerance in switchgrass by genetic transformation (Guan *et al*., 2018; Huang *et al*., 2017), the underlying molecular mechanisms remain largely unknown.

MicroRNA (miRNA), a kind of small non‐coding RNA with a typical length of ~21 nucleotides (nt), mediates target gene expression by mRNA cleavage at post‐transcriptional level or translational repression through base pairing with the complementary sequence within the target mRNAs (Axtell *et al*., 2007; Pasquinelli *et al*., 2000). MicroRNA319 (miR319) belongs to one of the most ancient and conserved miRNA families (Sun *et al*., 2012; Sunkar and Zhu, 2014). It has been shown that miR319 target to transcription factor TCP [TEOSINTE BRANCHED/CYCLOIDEA/PROLIFERATING CELL FACTORS (PCF)] genes (Nag *et al*., 2009; Schommer *et al*., 2008; Zhou *et al*., 2013) that were known to play important roles in plant development and responses to various stresses (Nag *et al*., 2009; Palatnik *et al*., 2003). Under salt stress, miR319 was found to be up‐regulated in many plants, including *A. thaliana* (Sunkar and Zhu, 2014), wheat (Wang *et al*., 2014), creeping bentgrass (Zhou *et al*., 2013) and switchgrass (Hivrale *et al*., 2016; Xie *et al*., 2014). Interestingly, salinity‐elicited down‐regulation of miR319 expression has also been reported in the roots of maize (Ding *et al*., 2009) and in *Solanum linnaeanum* (Kumar *et al*., 2018; Zhuang *et al*., 2014), suggesting a possible functional diversification of miR319 or its precursor in various plant species, different developmental stages and/or different tissues.

Overexpression of miR319 or repression of its targets, the *TCP* genes, was found to affect plant development (Nag *et al*., 2009; Palatnik *et al*., 2003; Schommer *et al*., 2014) and has led to improved plant tolerance to salt and drought stresses in transgenic creeping bentgrass (Zhou *et al*., 2013) and cold stress in rice (Yang *et al*., 2013). Up to now, one target of miR319, *PvPCF5*, has been identified experimentally in switchgrass (Xie *et al*., 2017). However, the function and mechanisms of miR319 or its target gene in the salt tolerance of this C4 grass species have yet to be revealed.

Ethylene, one of the essential signalling molecules, along with its downstream signalling components, was also reported to play a key role in salinity tolerance of rice and *A. thaliana* (Yang *et al*., 2015a; Zhang *et al*., 2016). Induced ethylene accumulation by endogenous production (Jiang *et al*., 2013) or exogenous application of 1‐aminocyclopropane‐1‐carboxylic acid (ACC), an ethylene precursor (Divi *et al*., 2010), could enhance *A. thaliana* salt tolerance by inducing downstream signalling to maintain reactive oxygen species (ROS; Peng *et al*., 2014; Yang *et al*., 2015b) and Na^+^ and K^+^ homeostasis (Jiang *et al*., 2013; Yang *et al*., 2015b). However, a negative role of ethylene in rice seedling tolerance to salt stress has also been reported (Ma *et al*., 2010; Tao *et al*., 2015). Overexpression of a wheat ethylene biosynthesis enzyme, ACC oxidase 1 (ACO1), enhanced ethylene levels but resulted in a salt‐sensitive phenotype (Chen *et al*., 2014). These data indicate ethylene negatively or positively regulates the plant response to salt stress (Zhang *et al*., 2016). The specific function of ethylene responding to salinity stress in different plant species associated with plant developmental stages remains largely unclear.

Phytohormone regulates miRNA expression in different plants (Chen *et al*., 2012; Duan *et al*., 2016; Liu *et al*., 2009). MiRNA‐mediated gene regulation has also been implicated in phytohormone crosstalk and signalling (Bai *et al*., 2017; Curaba *et al*., 2014; Liu *et al*., 2017). Under salt stress, a large number of miRNAs showed alternative expression in diverse plant species (Chen *et al*., 2012; Ding *et al*., 2009; Kumar *et al*., 2018). Among them, miR319 could be one of the master regulators because of the crucial role it plays in lateral organ development, senescence, responses to diverse stresses and implications in ethylene signalling (Curaba *et al*., 2014; Schommer *et al*., 2008; Zhang *et al*., 2016). MiR319 was reported to be down‐regulated in the root of *Medicago truncatula* 24 h after exogenous ACC treatment, suggesting a potential interaction of miR319 with ethylene (Chen *et al*., 2012). Recently, the miR319 non‐target TCP family gene *AtTCP5* (no miR319 target site exists in its mRNA sequence) in *A. thaliana* was reported to negatively regulate *ACS2*, the gene encoding a key ethylene biosynthesis enzyme, ACC synthase 2 (Van Es *et al*., 2018). In the dedicated monocot biofuel plant switchgrass, elucidating the molecular mechanism underlying the miR319‐mediated plant response to salt stress and the role of ethylene may play are of significant importance.

In this study, we aim to explore the function of the miR319‐*PvPCF5* module in switchgrass salt tolerance and investigate whether miR319 engages in crosstalk with ethylene to determine the plant response to salt stress. We produced three categories of transgenic switchgrass plants with OE‐miR319, inhibited miR319 (*MIM319*) by miRNA target *MIMICs* proven to be efficient in inhibiting miRNA function (Franco‐Zorrilla *et al*., 2007), which has been used to study the function of miR319 (Reichel and Millar, 2015; Todesco *et al*., 2010), and suppressed *PvPCF5* (a target of miR319) by overexpressing *PvPCF5‐SRDX* fusion gene using chimeric repressor gene‐silencing technology (Hiratsu *et al*., 2003). By testing the salt tolerance of transgenic switchgrass plants and their responses to salt stress when treated with ethylene or ACC, we demonstrate that the miR319‐*PvPCF5* module positively regulates the salt tolerance of switchgrass by promoting ethylene synthesis.

## Results

### MiR319 responds to salt stress and exogenous ethylene application in switchgrass

**Figure 1:**
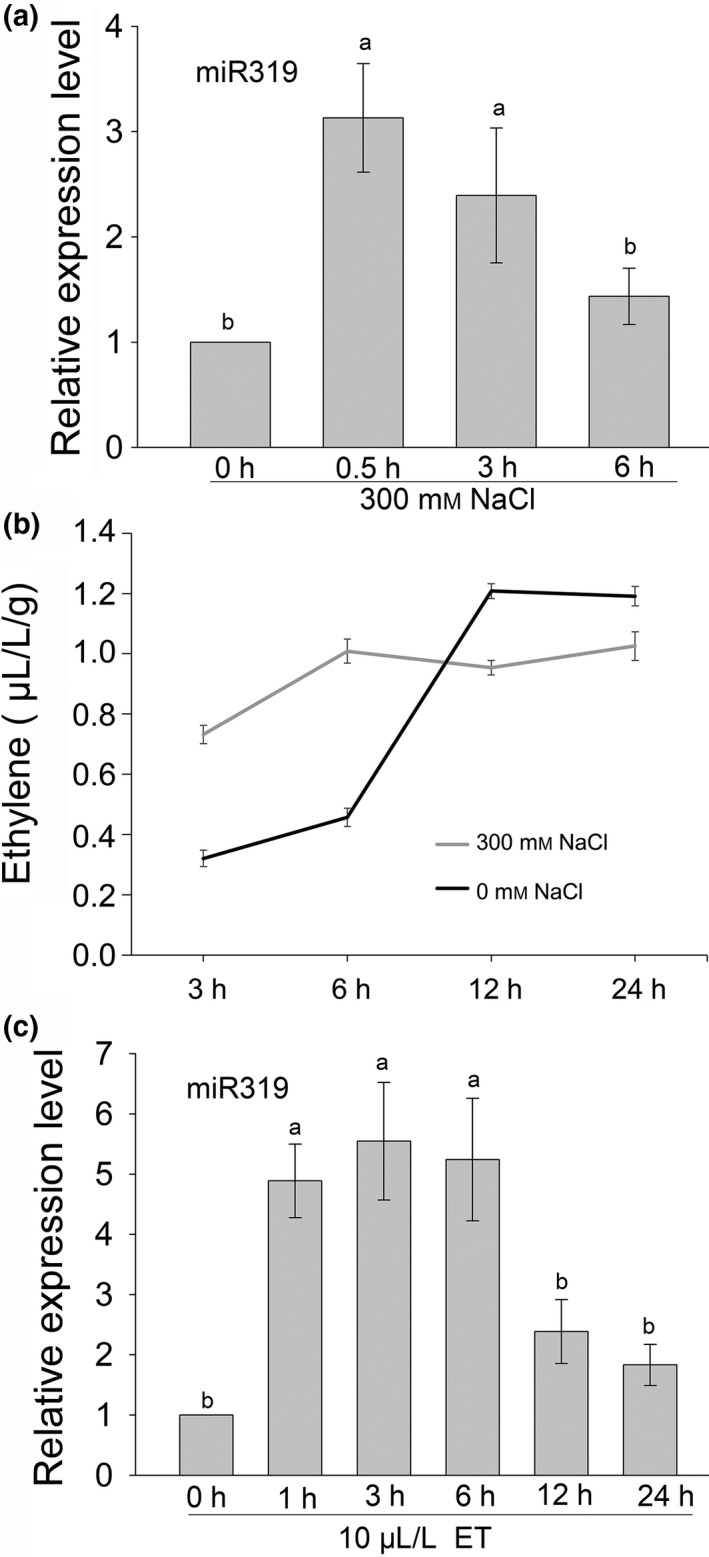
The accumulation of miR319 and ethylene production in leaves of E2 stage switchgrass plants under 300 mM NaCl stress and the effect of exogenous ethylene on the expression of miR319. Expression pattern of miR319 in leaves of switchgrass plants subjected to salt stress. (b) Ethylene production pattern of switchgrass detached leaves under NaCl‐free and 300 mM NaCl treatment. (c) Exogenous ethylene induced miR319 expression in 7‐day‐old switchgrass seedlings. A U6 snRNA was used as an internal control for mature miR319 analysis. The data are shown as the mean SD (independent biological samples, *N* = 3; technique repeats, n = 3 in a, c and *n* = 6 in b). Different letters indicate significant differences by one‐way *ANOVA* and Duncan's multiple comparisons post hoc analyses (*P* < 0.05).

To investigate whether miR319 plays a role in switchgrass salt response, we examined the miR319 accumulation level in switchgrass wild‐type (WT) E2 stage plants (Moore *et al*., 1991) subjected to 300 mm NaCl treatment. As shown in Figure 1a, the expression of mature miR319 in switchgrass leaves was significantly induced after 300 mm NaCl treatment for 0.5 h and then declined at 3 and 6 h but still remained at a higher level than that of the sample without treatment. We also tested ethylene production in switchgrass detached leaves subjected to salt stress and found that the detached leaves of switchgrass produced significantly more ethylene during treatment with 300 mm NaCl than with 0 mm NaCl at 3 and 6 h (Figure 1b). However, the detached leaves produced significantly more ethylene under 0 mm NaCl than under 300 mm NaCl 12 h after treatment (Figure 1b). To test whether exogenous ET would affect the expression of miR319 in switchgrass, 10 μL/L ET was applied to 7‐day‐old switchgrass seedlings and the miR319 expression level was shown to be significantly increased 1 h after treatment and remained at a significantly higher level until 6 h after treatment, then declined at 12 h (Figure 1c). The results indicate salt stress enhances miR319 accumulation at an early stage and promotes ethylene biosynthesis, while exogenous ethylene also induces miR319 expression in switchgrass.

### Generation of transgenic switchgrass plants with enhanced or inhibited miR319 expression

**Figure 2:**
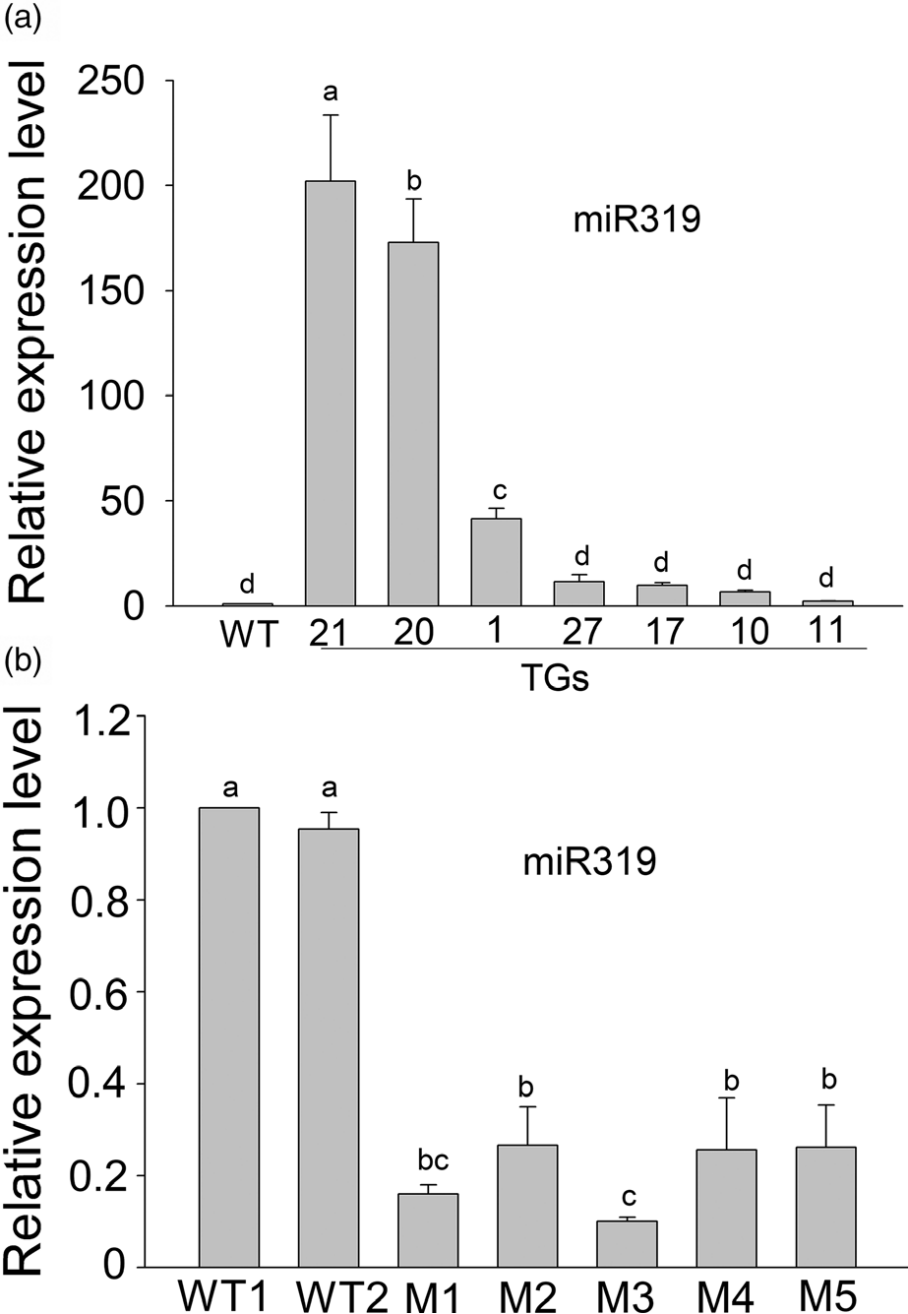
Real‐time qPCR tests the expression of miR319 in Osa‐miR319b‐overexpression plants (TGs) (a) and MIM319 transgenic plants (Ms) (b). A U6 snRNA was used as an internal control. The data are shown as the mean ± SD (*N* = 3, *n* = 3). Different letters indicate significant differences at *P* < 0.05.

To investigate the function of miR319 in switchgrass salt response, a rice *Osa‐MIR319b* gene and a *MIM319* generated from the *A. thaliana IPS* gene (Data S1; Figure S1a) were introduced into switchgrass. Positive transgenic plants were verified by PCR and RT‐PCR (Figure S1b, c, d and e). The accumulation of miR319 in the transgenic and WT plants was examined. OE‐miR319 lines TG20 and TG21, exhibiting significantly higher miR319 expression than that of the WT and other transgenic lines, were chosen for further analysis (Figure 2a). Target *MIMICs* are known to sequester their target miRNAs and affect miRNA activity and stability. In the *MIM319* transgenic plants, the abundance of miR319 was significantly reduced relative to that in WT controls (Figure 2b). The transgenic lines M1, M3 and M5 were chosen for further tests.

Discernible changes in phenotype associated with miR319 expression, such as leaf blade width, were observed in OE‐miR319 and *MIM319* (Ms) plants (Figure S2a, b). While the OE‐miR319 plants exhibited significantly wider leaves (approximately 4 mm) than those of the WT controls (Figure S2c), the leaf blade of the *MIM319* transgenics was significantly narrower (approximately 25%) than that of WT plants (Figure S2c). The dry biomass yield of the 6‐month‐old plants was significantly increased (approximately 37%) in OE‐miR319 plants but decreased (approximately 19%) in *MIM319* plants compared to that in WT controls (Figure S2d).

### MiR319 positively regulates plant salt tolerance in switchgrass

**Figure 3:**
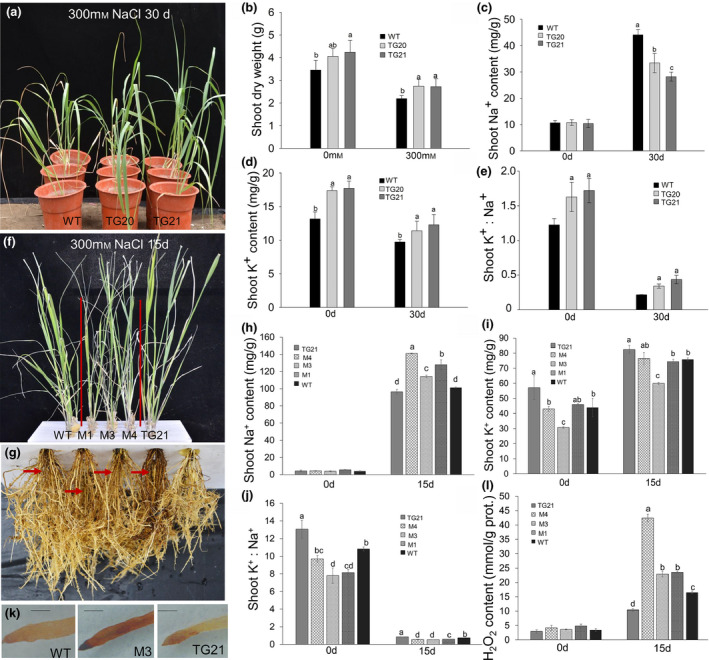
Salt tolerance tests of WT, OE‐miR319 and *MIM319* (Ms) switchgrass plants under 300 mm NaCl stress treatment in a silica‐culture system (a–e) and liquid‐culture system (f–l). (a) Under salt stress with 300 mM NaCl for 30 d, the TGs still showed healthy leaves, but the leaves of WT plants showed obvious chlorosis. (b) The shoot dry biomass was measured after salt treatment for 30 days. (c, d, e) The shoot Na^+^ and K^+^ contents (c, d) and K^+^/ Na^+^ ratio (e) before and after salt treatment for 30 d. (f, g) Comparison of shoot (f) and root (g) performance of wild‐type and transgenic plants subjected to 300 mM NaCl salt stress treatment for 15 d. The red arrows indicate browning and necrotic roots of WT and Ms plants (g). (h, i, j) The shoot Na^+^ (h) and K^+^ (i) contents and the K^+^/Na^+^ ratio (j). (k) DAB staining assay of the root‐tips of WT, M3 and TG21 plants; the stronger brown colour indicates higher accumulation of H_2_O_2_. The length of the scale bar represents 1 mm. (l) Quantitative analysis of H_2_O_2_ content in roots before and after 15 d of salt stress treatment. The data are shown as the mean ± SD (*N* = 3, *n* = 3). Different letters indicate significant differences by one‐way *ANOVA* and Duncan's multiple comparisons post hoc analysis (*P* < 0.05).

To investigate how OE‐miR319 impacts the salt response in transgenic switchgrass plants, we first examined the salt tolerance of WT and two OE‐miR319 lines (TG20 and TG21) at the E2 stage in a silica‐culture condition (Figure S3a). As shown in Figure 3a, after treatment with 300 mm NaCl for 30 days, TG20 and TG21 exhibited much better phenotypes with greener and normal leaves than those of WT controls displaying obvious chlorosis and curling leaves. The above‐ground biomass yield of TG20 and TG21 plants was approximately 17% and 28% more than that of WT plants under 0 mm salt treatment and 300 mm NaCl for 30 days, respectively (Figure 3b). However, the root biomass of WT and OE‐miR319 plants showed no significant difference before and after 300 mm NaCl treatment (Figure S3b). The shoot and root Na^+^ contents showed no significant differences between OE‐miR319 and WT plants before salt treatment. However, after salt treatment for 30 days, the WT plants accumulated markedly more Na^+^ in both the shoots and roots than transgenic plants. Of all the plants tested, TG21 had the lowest Na^+^ accumulation (Figures 3c, S3c). Before salt treatment, the K^+^ content of OE‐miR319 plants was approximately 5 mg/g higher than that of WT plants (Figures 3d, S3d). After salt treatment for 30 day, although the K^+^ content of both WT and OE‐miR319 plants decreased compared to that at 0 day, the K^+^ content in OE‐miR319 plants remained significantly higher than that in WT controls (Figures 3d, S3d). The higher K^+^ and lower Na^+^ contents of the OE‐miR319 plants also resulted in a significantly higher K^+^/Na^+^ ratio than that of WT controls in both the shoots and roots (Figures 3e, S3e).

Next, we tested the salt tolerance of E2 stage switchgrass plants cultured in 1/4 Hoagland's nutrient solution supplied with 300 mm NaCl (Figure S3f). Upon 300 mm NaCl treatment for fifteen days, the leaves of *MIM319* plants (M1, M3 and M4) exhibited obvious wilting and chlorosis compared to that of the WT and TG21 plants (Figure 3f). The roots of the *MIM319* plants (especially M4) showed brown colour compared to those of WT plants, whereas the roots of TG21 were much healthier than those of WT plants (Figure 3g). In the liquid‐culture condition, the plants obviously absorbed Na^+^ much faster than in sand culture. As shown in Figure 3h, the shoot Na^+^ content in the liquid‐culture condition for 15 day was remarkably higher (the lowest was 100 mg/g in TG21) than that in the sand‐culture condition for 30 days (the highest was 45 mg/g in WT; Figure 2c). The Na^+^ content in the roots of *MIM319* plants was also significantly higher than that of WT and TG21 plants (Figure S3g). Under Na^+^‐free conditions, the K^+^ content in the roots and shoots of the OE‐miR319 line TG21 was significantly higher than that of WT and *MIM319* plants (Figures 3i, S3h). After 300 mm NaCl treatment for 15 day, the K^+^ content in the roots of TG21 and WT plants increased to 16 and 13 mg/g, respectively; these values were approximately two times the K^+^ content in the roots of the *MIM319* plants (Figure S3h). In shoots, the K^+^ content of TG21 was significantly higher than that of WT and *MIM319* plants. However, the K^+^ content of WT plants was only significantly higher than that of M3 plants (Figure 3i). The lower K^+^ and higher Na^+^ content in *MIM319* plants resulted in a significantly lower K^+^/Na^+^ ratio than that in TG21 and WT plants before and after salt treatment (Figures 3j, S3i). In a DAB staining assay, the root tip of the *MIM319* line M3 showed much stronger brown colour than that of WT and TG21 plants (Figure 3k). The root H_2_O_2_ content of the tested plants showed no significant difference before salt treatment, but significantly increased 15 day after salt treatment in all tested plants (Figure 3l). The amounts of root H_2_O_2_ accumulation in *MIM319* plants were all significantly higher than in WT controls. The lowest root H_2_O_2_ accumulation was observed in TG21 (Figure 3l). These results indicated miR319 positively regulates salt tolerance in switchgrass via increased plant K^+^ uptake and/or Na^+^ exclusion and enhanced scavenging of ROS generated under salt stress.

### MiR319 positively regulates ethylene biosynthesis in switchgrass plants

**Figure 4:**
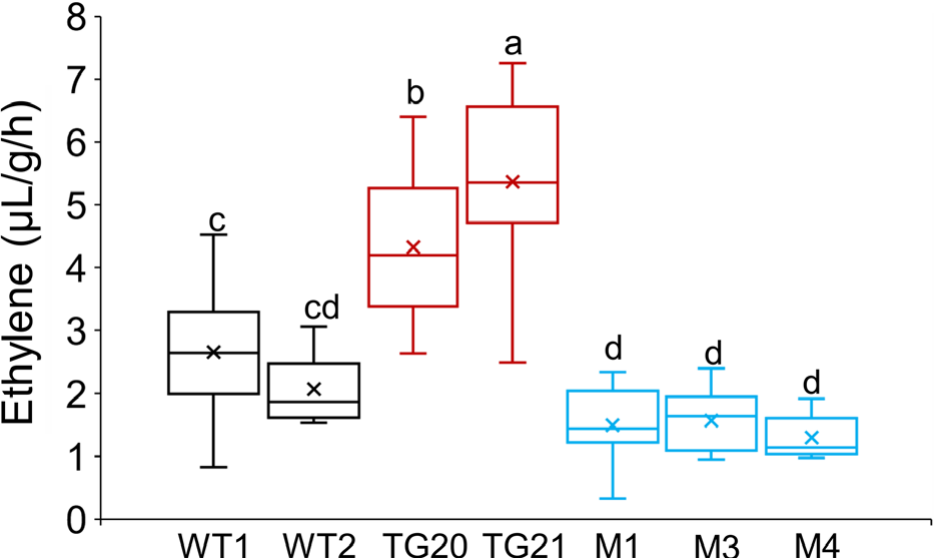
MiR319 positively regulates ethylene synthesis in switchgrass plants. Comparison of ethylene production in the detached leaves of WT, OE‐miR319 (TG20, TG21) and *MIM319* (M1, M3, M4) plants. The center lines show the medians, “×” shows the mean value, box limits indicate 25% to 7% of the values as determined by R software, the down and up whiskers extended to the minimum and maximum values (*n* = 18). Different letters indicate significant differences by one‐way *ANOVA* and Duncan's multiple comparisons post hoc analysis (*P* < 0.05).

To explore the effect of altered miR319 expression on ethylene synthesis, we measured ethylene production in the detached leaves of the WT, OE‐miR319 and *MIM319* plants. The results showed that the ethylene production of OE‐miR319 plants was significantly higher than that of WT and *MIM319* plants (Figure 4). The transgenic line TG21 had higher expression of miR319 than TG20, and its ethylene production was also significantly higher than that of TG20. Clearly, overexpression of miR319 enhanced ethylene synthesis in switchgrass.

### A low dose of exogenous ethylene or ACC enhances salt tolerance of switchgrass seedlings

To investigate the effect of ET on the switchgrass response to salt stress, we treated germinated (2 day) WT switchgrass seeds with 150 mm NaCl combined with different concentrations of ET for 7 or 15 days. The results showed that the growth of seedlings was obviously hindered with curly leaves following treatment without ET or treatment with an ET concentration over 4.0 μL/L (Figure S4a). After salt treatment combined with different concentrations of ET for 7 day or 15 day, the seedlings treated with 10 μL/L ET consistently showed even shorter shoots than those treated with 0.0 μL/L ET. However, when the seedlings were treated with a lower concentration of ET, which increased ET content from 0.5 to 2.0 μL/L, shoot length was significantly increased, and shoot length declined when ET content was over 4.0 μL/L (Figure S4a, b). Examining the H_2_O_2_ contents of seedlings treated with various amounts of ET revealed that the seedlings treated with 2.0 μL/L ET accumulated the lowest H_2_O_2_ at the two tested time points (Figure S4c). Exogenous ET treatment led to less H_2_O_2_ accumulation in the seedlings than in non‐treated controls (Figure S4c).

**Figure 5:**
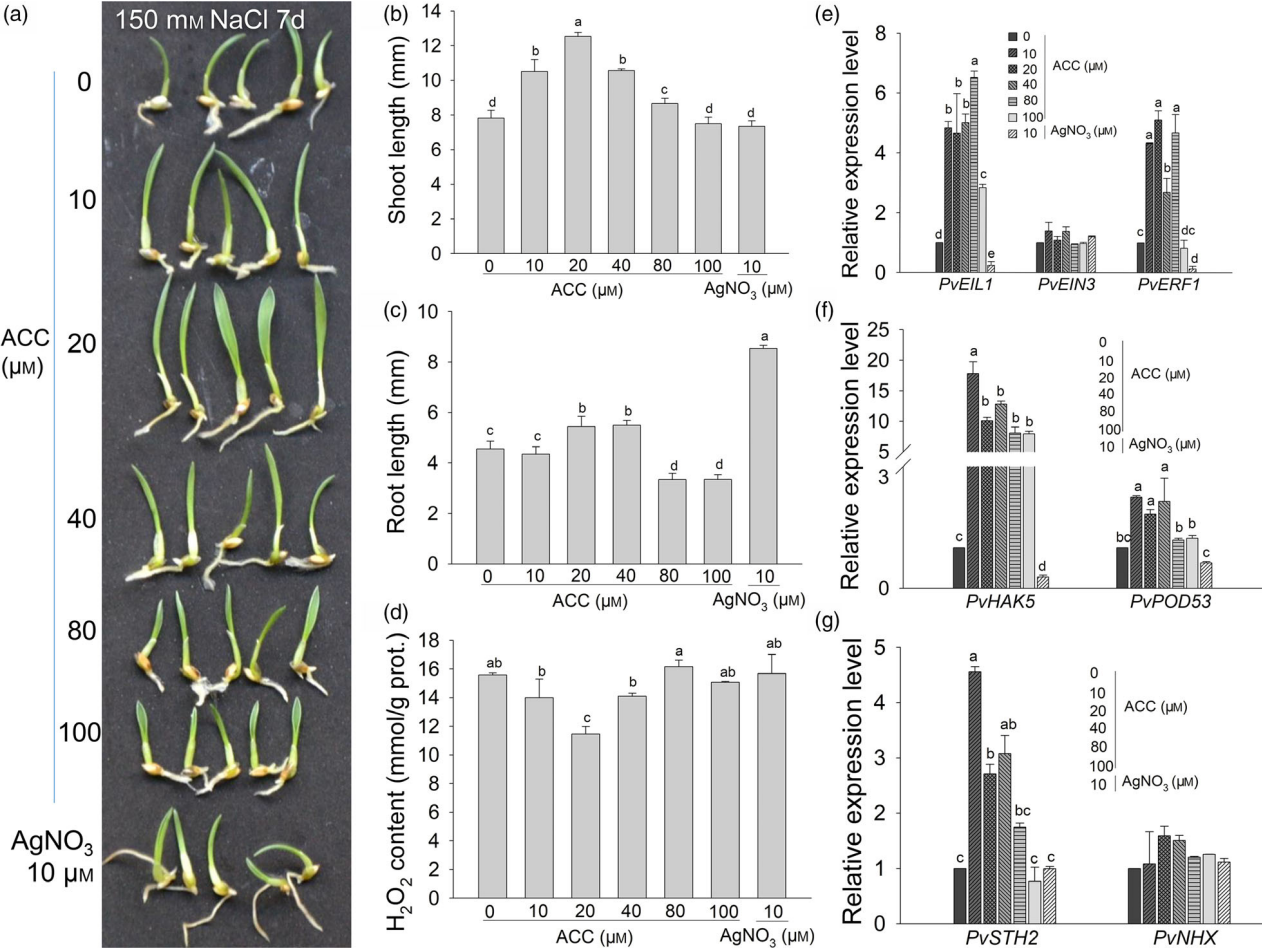
Dose‐dependent effects of the additional ethylene precursor ACC on salt stress and the expression of genes related to ethylene signalling in WT switchgrass seedlings.(a) The phenotype of switchgrass WT seedlings germinated under 150 mM NaCl stress treatment in combination with additional different concentrations of ACC or 10 μM AgNO3 for 7 d. (b, c, d) Shoot lengths (b), root lengths (c) and H_2_O_2_ accumulation of the germinated seedlings were measured after treatment (d). (e, f, g) Comparison of gene expression related to the ethylene signaling pathway (e), K^+^ absorption and reactive oxygen species scavenging (f) and salt tolerance (g) in the germinated seedlings after treatment for 7 d. The data are shown as the mean ± SD (*N* = 3, *n* = 3). Twenty (b, c, d) and ten (e, f, g) seedlings were sampled as one replicate. Different letters indicate significant differences by one‐way *ANOVA* and Duncan's multiple comparisons post hoc analysis (*P* < 0.05). *PvEIL1*: switchgrass ethylene insensitive 3‐like 1; *PvEIN3*: switchgrass ethylene insensitive 3; *PvERF1*: ethylene response factor 1; *PvHAK5*: high affinity K^+^ transporter 5; *PvPOD53*: peroxidase 53; *PvSTH2*: *SALT TOLERANCE HOMOLOGE* 2; PvNHX: Na^+^/H^+^ exchanger.

To further verify the dose‐dependent effects of ET on switchgrass salt tolerance, we also treated germinated (2 day) WT switchgrass seeds with 150 mm NaCl combined with different concentrations of the ethylene precursor ACC or 10 μm AgNO_3_ to inhibit ethylene action in the liquid culture for 7 days. The results showed that the shoot length steadily increased to the longest length following treatment with 20 μm ACC, but then declined when the ACC concentration was over 20 μm (Figure 5a, b). The seedlings treated with 10 μm AgNO_3_ had the longest roots, followed by those treated with 20 and 40 μm ACC, whose roots were significantly longer than those treated with 0 and 10 μm ACC (Figure 5a, c). Compared to 0 μm ACC treatment, when ACC content was over 80 μm, root elongation was significantly inhibited (Figure 5c). Seedlings treated with 20 μm ACC also accumulated significantly lower amount of H_2_O_2_ than others after 7 day of combined treatment. However, in the other treatment groups, the H_2_O_2_ content was significantly higher than that following treatment with 20 μm ACC (Figure 5d).

To explore the possible mechanisms of the ET‐ or ACC‐mediated dose‐dependent salt tolerance in switchgrass, the expression levels of seven switchgrass homologous genes associated with ET signalling transduction (*PvEIL1, PvEIN3*), ET responding factors (*PvERF1, PvHAK5, PvPOD53*) and salt tolerance genes (*PvSTH2, PvNHX*; Table S2) were analysed in switchgrass seedlings subjected to combined treatments with 150 mm NaCl and different concentrations of ACC or AgNO_3_ for 7 day (Figure 5e, f, g). Except for *PvEIN3* and *PvNHX,* the expression of all other genes was significantly induced by lower levels of ACC (10–40 μm) but tended to decline at higher levels of ACC (80–100 μm). The expression levels of *PvERF1*,* PvPOD53* and *PvSTH2* showed no significant differences between WT plants treated with exogenous 100 μm ACC and those under ACC‐free conditions (Figure 5e, f, g). Following treatment with 10 μm AgNO_3_, the expression of *PvERF1*,* PvEIL1*,* PvHAK5* and *PvPOD53* was considerably reduced compared to that following treatment with 0 μm ACC (Figure 5a, e, f, g). These results indicate that a lower dosage of exogenous ACC enhanced switchgrass salt tolerance by inducing ethylene signalling and downstream gene expression.

### MiR319 improves the salt tolerance of switchgrass by enhancing ET biosynthesis

**Figure 6:**
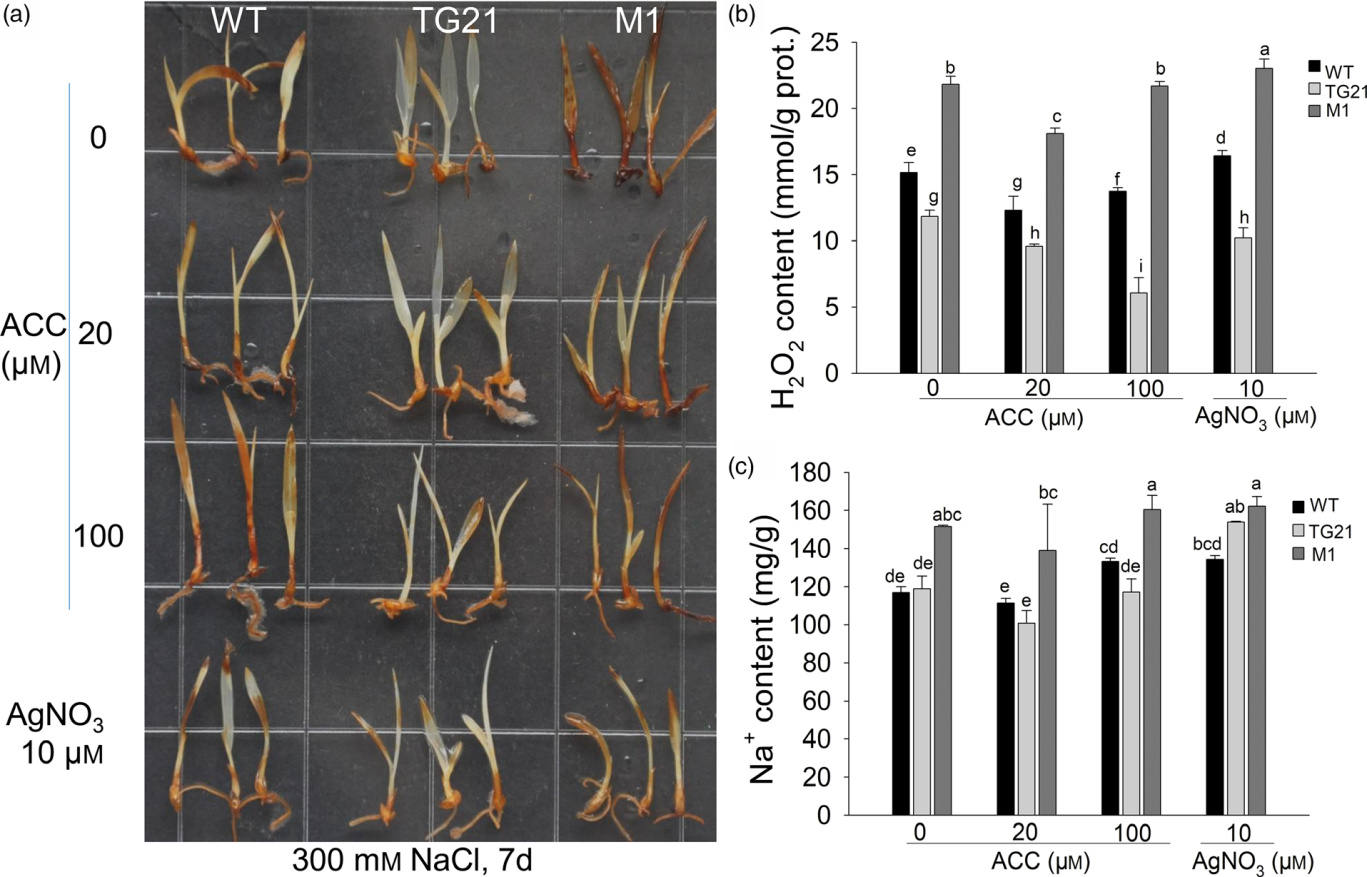
Comparison of the effects of exogenous ACC and AgNO_3_ on the salt tolerance responses of switchgrass 7‐day‐old seedlings. (a) DAB staining to test the accumulation of H_2_O_2_ in 7‐day‐old WT, TG21 and M1 seedlings under combined treatments for 7 d. (b) Quantitative analysis of H_2_O_2_ accumulation in the seedlings. (c) Na^+^ content test in the seedlings. The data are shown as the mean ± SD (*N* = 3, *n* = 3). Twenty seedlings (b, c) were sampled as one replicate. Different letters indicate significant differences by one‐way *ANOVA* and Duncan's multiple comparisons post hoc analysis (*P* < 0.05).

We also compared the phenotypes of 7‐day‐old WT, TG21 and M1 seedlings under 300 mm NaCl treatment combined with different concentrations of ACC (0, 20, 100 μm) or 10 μm AgNO_3_ for 7 days. To reveal H_2_O_2_ accumulation in the seedlings due to salt stress, a DAB staining assay was carried out. Stronger brown colour indicates a higher level of H_2_O_2_ accumulation. The results showed that the brown colour displayed in the WT seedlings was stronger than in the OE‐miR319 TG21‐line T_1_ seedlings, but lighter than in the M1 seedlings under the same treatments (Figure 6a). The seedlings cultured with 20 μm ACC displayed noticeably lighter brown colour in the DAB staining assay than those cultured in other treatments (Figure 6a). Measurement of H_2_O_2_ and Na^+^ content revealed that M1 seedlings accumulated significantly higher H_2_O_2_ and Na^+^ than WT and TG21 seedlings under all treatments, except 10 μm AgNO_3_, for which the Na^+^ content of M1 showed no significant difference from that of TG21 but was still higher than that of the latter (Figure 6b, c). H_2_O_2_ content in TG21 was significantly lower than in WT and M1 and showed a decreasing tendency with the increasing concentrations of ACC. However, the lowest H_2_O_2_ contents in WT and M1 seedlings were observed following treatment with 20 μm ACC (Figure 6b). The Na^+^ contents of WT and TG21 seedlings showed no significant difference but were significantly lower than that of M1 under the same ACC treatment (Figure 6c).

### Identification of miR319 putative target genes and their responses to ethylene and salt stress

**Figure 7:**
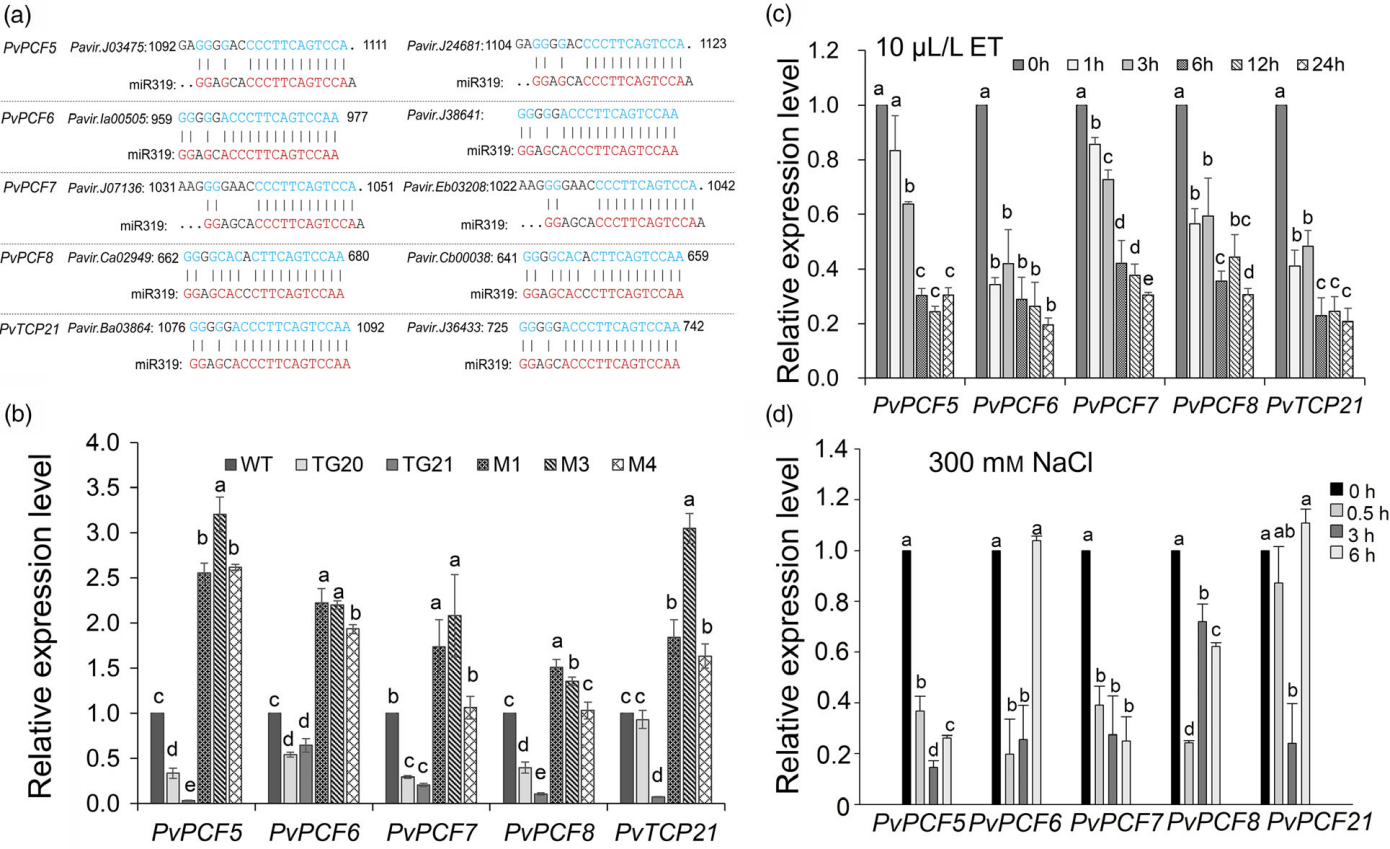
Analysis of the miR319 binding sites in *PvTCPs* and measurement of their expression. (a) The predicted miR319 target sites in the five *PvTCP* genes. (b) qRT‐PCR analysis of *PvTCP*s expression in WT, OE‐miR319 (TGs) and *MIM319* (Ms) plants. (c, d) Temporal expression pattern of *PvTCPs* under 10 L L^‐1^ ET treatment (c) or 300 mM salt treatment (d) in 7‐day‐old WT seedlings. The data are shown as the mean ± SD (*N* = 3, *n* = 3). Different letters indicate significant differences by one‐way ANOVA and Duncan's multiple comparisons post hoc analysis (*P* < 0.05).

It has been noted that miR319 down‐regulates its target genes by complementary base pairing to exert its biological function (Yang *et al*., 2013; Zhou *et al*., 2013). In this study, sequences of putative miR319 target genes in switchgrass were obtained from the database of https://phytozome.jgi.doe.gov/by BLASTN searches with the homologous sequences of *TCP* genes, the miR319 targets in rice and *A. thaliana* (Figure S5). Five *PvTCP* genes, *PvPCF5*,* 6*,* 7*,* 8* and *PvTCP21*, with two isoforms each defined as ‘a’ and ‘b’, were identified, and the miR319 binding site is shown in Figure 7a. Phylogenetic analysis showed that the amino acid sequences of *TCPs* were conserved among switchgrass, *A. thaliana* and rice (Figure S5). Quantitative real‐time PCR (qRT‐PCR) analyses showed that the expression of the five *PvTCPs* was down‐regulated in OE‐miR319 lines, but significantly up‐regulated in *MIM319* plants (Figure 7b), indicating the five *PvTCPs* were negatively regulated by miR319. To analyse whether *PvTCPs* are involved in salt stress responses, we tested the expression of *PvTCPs* under ET and salt stress treatments. When switchgrass WT plants were treated with 10 μL/L ET, the expression level of the five *PvTCPs* showed a significant down‐regulation of the tested time points within 24 h (Figure 7c). Under 300 mm NaCl treatment, the expression of *PvTCPs* steadily decreased at 0.5 and 3 h and then increased at 6 h, except for *PvPCF7* (Figure 7d). The results suggest that the expression of the tested *PvTCPs* responds to ethylene and salt stress.

### Repression of the miR319 target gene *PvPCF5* improves ET synthesis and salt tolerance

**Figure 8:**
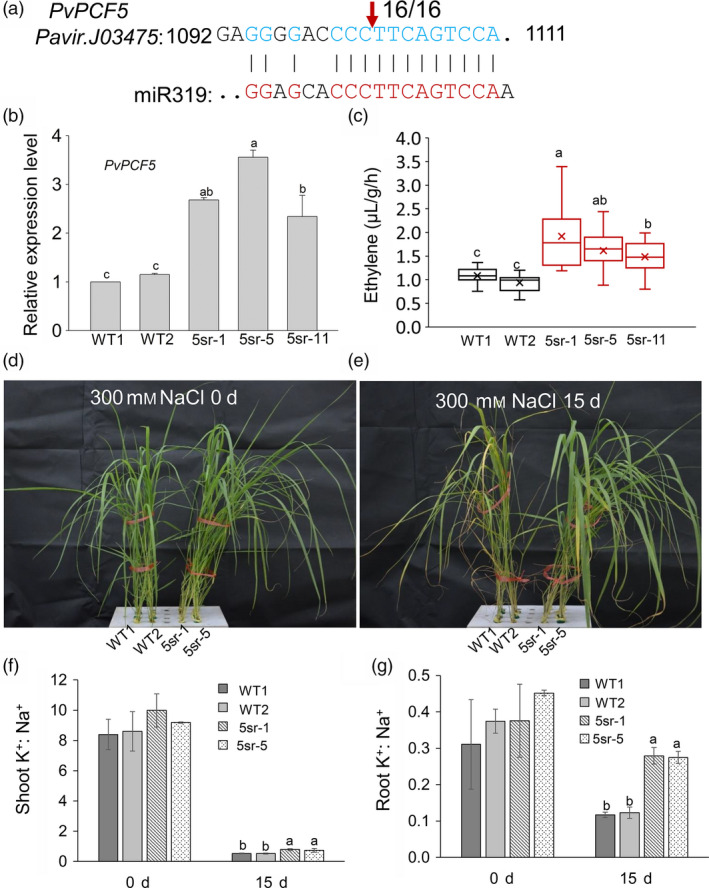
Repression of the validated miR319 target *PvPCF5* enhanced the salt tolerance of switchgrass transgenic plants. (a) Analysis of the miR319 cleavage site in PvPCF5 by 5′ RLM‐RACE, indicated by the vertical arrow with the frequency of clones shown. (b) qRT‐PCR verification of PvPCF5 expression in PvPCF5‐SRDX transgenic plants (5sr). (c) Ethylene production assay of the isolated leaf of WT and PvPCF5‐SRDX (5sr) transgenic plants. The center lines show the medians, “×” shows the mean value, box limits indicate 25% to 75% of the values as determined by R software, the down and up whiskers extend to the minimum and maximum values (n = 18). (d, e) The state of WT and 5sr plants before (d) and after 300 mM NaCl salt stress treatment for 15 d (e). (f, g) The K+/Na+ ratio in shoots (f) and roots (g) before and after salt stress treatment for 15 d. The data are shown as the mean ± SD (N = 3, n = 3 in b, f and g). Different letters indicate significant differences by one‐way ANOVA and Duncan's multiple comparisons post hoc analysis (*P* < 0.05).

To experimentally prove the function of *PvPCFs* in salt tolerance and ethylene synthesis, *PvPCF5* (Pavir.J03475) was cloned for further study. We verified the miR319 cleavage site in *PvPCF5* using a 5′ RNA ligase‐mediated (RLM) rapid amplification of cDNA ends (RACE) assay. The results showed the transcript of *PvPCF5* was cleaved between base pairs 10 and 11 of the miR319 target site (Figure 8a). We suppressed the function of *PvPCF5* by linking it to the EAR‐motif repression domain SRDX (Figure S6a), creating a dominant repressor that could inhibit the expression of its target genes. The *PvPCF5‐SRDX* transgenic plants would show a phenotype similar to that of *PvPCF5* loss‐of‐function mutants. Transgenic plants overexpressing *PvPCF5‐SRDX* were verified by PCR (Figure S6b) and qRT‐PCR tests (Figure 8b). Three *PvPCF5‐SRDX* (5sr) transgenic lines (5sr‐1, 5sr‐5 and 5sr‐11) that showed 2–4 times higher expression of *PvPCF5* than that of WT plants in qRT‐PCR tests were then chosen for salt tolerance tests (Figure 8b). 5sr transgenic lines were taller with obviously wider leaves (Figure S7a, b, c) and consequently producing significantly more biomass than WT plants (Figure S7d).

To explore whether repressed *PvPCF5* function also enhances ET synthesis, we examined the ET production of 5sr lines (5sr‐1, 5sr‐5 and 5sr‐11). The results showed that the ET production of the three tested 5sr lines was approximately twofold that of WT plants (Figure 8c). To evaluate the salt tolerance of 5sr lines, E2 stage WT and 5sr plants (5sr‐1, 5sr‐5) were cultured in 1/4 Hoagland's nutrient solution containing 300 mm NaCl for 15 day (Figure 8d, e). The phenotypes of the 5sr plants were comparable, and their leaves were greener with fewer chlorotic lesions than those of WT plants (Figure 8e). Na^+^ accumulation in 5sr roots and shoots was significantly lower than that of WT plants (Figure S8a, b). The roots of 5sr plants accumulated significantly more K^+^ than those of WT plants after salt treatment (Figure S8c), and shoot K^+^ contents showed no significant difference between WT and 5sr before and after salt treatment (Figure S8d). A significantly higher K^+^/Na^+^ ratio in the roots and shoots of the 5sr plants than that of WT plants was observed 15 day after salt treatment (Figure 8f, g). The results indicate that *PvPCF5* negatively regulates ET synthesis and salt tolerance in switchgrass.

### Modified expression of miR319 alters the transcriptome associated with stress regulation, especially in the ET biosynthesis pathway

To explore the potential molecular mechanisms of improved salt tolerance and ET synthesis in OE‐miR319 plants, RNA sequencing was performed to analyse the transcriptome of the WT and OE‐miR319 lines (TG21 and TG20). The genes with adjusted *P*‐values < 0.05 were identified as differentially expressed genes (DEGs). The results showed 12253 DEGs between TG and WT plants (Figure S9a), including 6757 up‐regulated and 5494 down‐regulated genes. The abundance of DEGs with different log2 fold change (FC) values is shown in Figure S9b. There were 874 DEGs showing significant changes in expression (absolute value of log2 FC ≥ 3), including 232 down‐regulated and 642 up‐regulated genes. Functional annotation of significantly regulated genes revealed that overexpression of miR319 affected multiple biological processes related to plant growth and stress responses, including chloroplast and plastid development, peroxidase reaction, and membrane process, among others (Figure S10a, b). To validate the RNA‐seq data, the expression of 10 randomly selected DEGs was examined by qRT‐PCR and found to be consistent with that determined by RNA‐seq (Figure S10c, d).

**Figure 9:**
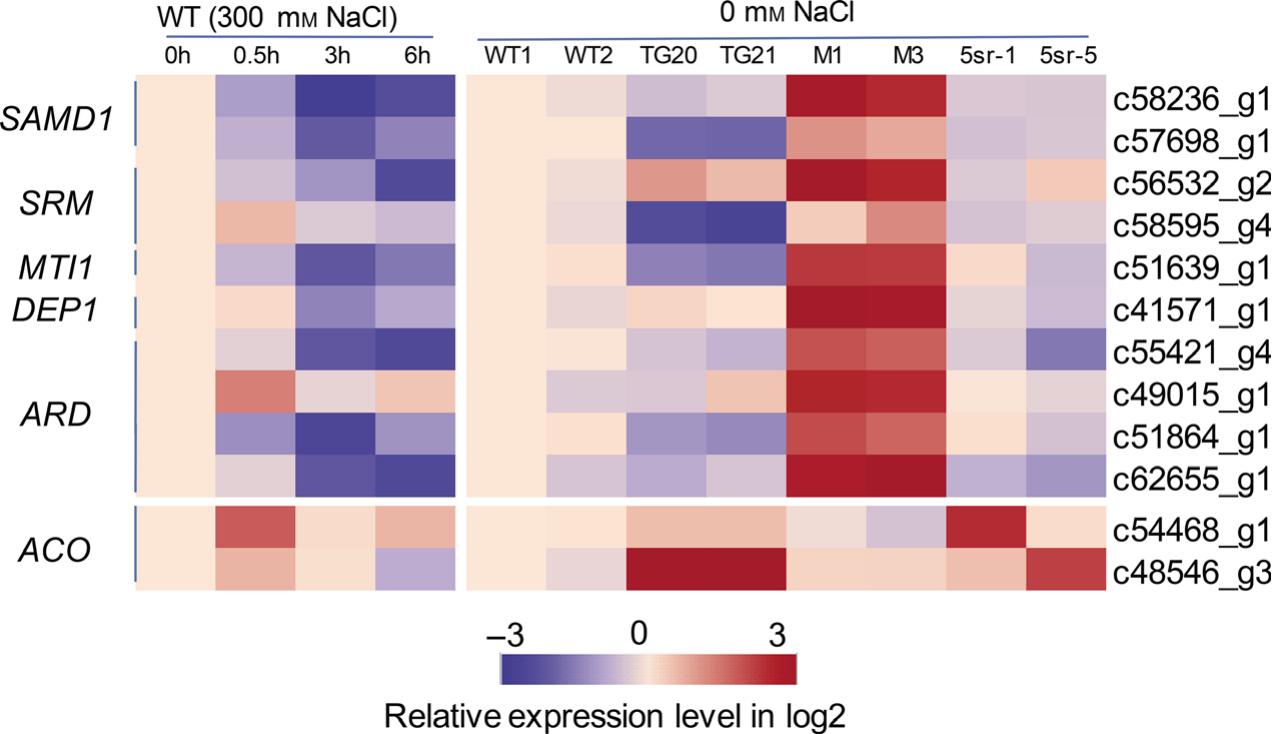
Heat map of the relative expression levels of genes related to the methionine (Met) salvage cycle and ethylene synthesis (ACO) in the leaves of WT plants treated with 300 mm NaCl and in the leaves of WT, OE‐miR319 (TG20, TG21), *MIM319* (M1, M3, M4) and *PvPCF5‐SRDX* transgenic plants (5sr‐1, 5sr‐5) grown under NaCl‐free conditions. The relative expression levels of genes were tested by qRT‐PCR based on log2‐fold change (*N* = 3, *n* = 3). SAMD1: S‐adenosylmethionine decarboxylase1; SRM: spermidine synthase; *MTI1*: 5‐METHYLTHIORIBOSE‐1‐PHOSPHATE ISOMERASE1; DEP1: DEHYDRATASE‐ENOLASE‐PHOSPHATASE‐COMPLEX1; *ARD*: acidoreductone oxygenase; ACO, 1‐aminocyclopropane‐1‐carboxylic acid (ACC) oxidases.

Regarding the function of miR319 in switchgrass, we were particularly interested in genes annotated as being involved in ethylene synthesis and signalling and the abiotic stress response. As shown in Table S3, nineteen DEGs were found to be related to ethylene biosynthesis, of which three encoding the ACO1 enzyme were up‐regulated, and twelve methionine salvage genes were all significantly down‐regulated in OE‐miR319 lines. Most genes annotated to *EIN3/EIL1* were significantly up‐regulated in OE‐miR319 lines, and many genes in EIN3‐dependent ethylene signalling, such as *ERFs*,* HKTs* and *PODs*, changed their transcriptional levels in OE‐miR319 plants (Table S3). To evaluate whether ethylene biosynthesis genes respond to salt stress in switchgrass, the *ACOs* and methionine salvage‐related genes (*MSGs*) whose expression was significantly different between TG21 and WT by transcriptome analysis were analysed by qRT‐PCR in WT plants at 0 h, 0.5 h, 3 h and 6 h after treatment with 300 mm NaCl (Figure 9). The results showed that the tested *MSGs* were all down‐regulated by salt stress, but the expression of the two *ACOs* increased 0.5 h after treatment and then declined. Furthermore, the expression of *MSGs* was down‐regulated in the OE‐miR319 lines TG20 and TG21, consistent with the transcriptome analysis results (Table S3), and up‐regulated in M1 and M3 compared with WT plants (Figure 9). Expression of the *MSGs* was also down‐regulated in the 5sr transgenic lines. The expression of *ACOs* in OE‐miR319 lines was significantly up‐regulated compared to that in WT plants, but there was no significant difference between *MIM319* and WT plants. The two tested *ACO* genes exhibited up‐regulated expression in 5sr‐1 and 5sr‐5. The results suggest that OE‐miR319 enhances ethylene synthesis through repression of its target gene *PvPCF5* and then down‐regulation of *MSGs* and up‐regulation of *ACOs* (Figure 9). We also observed altered expression of ethylene‐independent salt‐related genes in OE‐miR319 lines, such as ion transporter‐encoding genes (e.g. *NHX*,* VCX1*) and transcription factors (e.g. NFYC, HSP20, MADS, MYB; Table S3), which had been implicated in the plant salt response (Chen *et al*., 2015; Guo *et al*., 2016; He *et al*., 2012; Huang *et al*., 2017; Sato and Yokoya, 2008). These results indicate that the molecular mechanisms of the miR319‐mediated salt response in switchgrass are complicated and may involve both ethylene‐dependent and ethylene‐independent pathways.

## Discussion

Previous reports indicated OE‐miR319 could successfully improve C3 plant salt tolerance by regulating multiple physiological pathways (Zhou *et al*., 2013; Zhu, 2002). However, the function of miR319 and its target *TCP* genes has barely been reported in C4 plants, and the mechanisms of miR319‐*TCPs* module‐enhanced plant resistance to abiotic stresses have not been reported. In this study, we demonstrate that the miR319‐*PvPCF5* module positively regulates salt tolerance by engaging the ethylene biosynthesis pathway in switchgrass, a dedicated C4 bioenergy plant. Through transcriptome analysis, we revealed many meaningful DEGs annotated to abiotic stress resistance, of which a plethora of DEGs were related to ethylene biosynthesis, ET signalling transduction and downstream response pathways. We speculate the regulation of ET biosynthesis and signalling by the miR319‐*PvPCF5* module mainly contributes to enhanced salt tolerance in switchgrass.

In this report, the switchgrass leaves showed enhanced ethylene biosynthesis levels during the first six hours under salt stress. It has been known that ethylene biosynthesis and signalling play essential roles in plant salt tolerance (Bürstenbinder *et al*., 2007; Zhang *et al*., 2016). Previous reports showed that elevated endogenous ethylene content in mutants or added ACC *in vitro* could increase *A. thaliana* salt resistance (Bürstenbinder *et al*., 2007; Divi *et al*., 2010). However, overexpression of a wheat *ACO1* gene in *A. thaliana* up‐regulated ET levels, and transgenic plants displayed a salt‐sensitive phenotype (Chen *et al*., 2014). In this study, we demonstrate that the enhanced salt tolerance in switchgrass induced by the application of exogenous ET or ACC is dose‐dependent. Lower doses of ET (0.5–4.0 μL/L) or ACC (10–40 μm) significantly enhanced salt tolerance in switchgrass seedlings. Over‐dosage of ACC or ET negatively affected salt tolerance in WT switchgrass seedlings, but had less impact on OE‐miR319 switchgrass seedlings. This phenomenon may be explained by miR319‐regulated expression of salt tolerance‐related genes both in and out of ethylene signalling pathways.

In the biosynthesis of ET, nicotianamine and polyamine produce a by‐product, 5‐methylthioadenosine (MTA), which is recycled to Met through a chain‐catalysed reaction by six enzymes (MTN1/2, MTK, MTI, ARD1, TAT and MAT) in plants; this process is called the Met cycle (Bürstenbinder *et al*., 2007; Waduwara‐Jayabahu *et al*., 2012; Zierer *et al*., 2016). *A. thaliana* mutants of the Met cycle were found to synthesize higher levels of ET when grown in darkness (Bürstenbinder *et al*., 2007; Woeste *et al*., 1999). We found that the miR319‐*PvPCF5* module negatively regulated the expression of the key genes in the Met cycle in switchgrass but significantly promoted the expression of at least certain *ACO* genes, explaining the higher ET levels in the detached leaves of OE‐miR319 and *PvPCF5‐SRDX* plants. Recently, a *TCP5* gene in *A. thaliana* was reported to be able to directly bind to the ethylene biosynthesis gene *ACS2* to inhibit ethylene biosynthesis (Van Es *et al*., 2018), an example demonstrating the possible involvement of TCP family members in regulating ethylene biosynthesis.

Improved ET biosynthesis was also accompanied by enhanced ET signalling transduction. EIN3 (ethylene insensitive 3) and EIL1 (EIN3‐like 1) are two ethylene‐activated transcription factors that reportedly to play a role in plant salt tolerance by increasing POD expression to preclude excess ROS accumulation (Peng *et al*., 2014; Yang *et al*., 2015b). The expression of *EIN3* and *EIL1* genes in the switchgrass OE‐miR319 line TG21 was significantly up‐regulated, and the expression levels of 28 *POD* genes were significantly increased, which could contribute to increased salt tolerance in TG21 by reducing ROS accumulation. Enhanced ET signalling transduction could also activate the expression of high‐affinity potassium transporters, which play an important role in K^+^ uptake (Jiang *et al*., 2013; Yang *et al*., 2015b). Compared to WT plants, five out of seven annotated K^+^ transporter genes, including *HAK*,* HKT* and *AKT*, in OE‐miR319 lines were found to be up‐regulated in the RNA sequencing data, especially the *HAK* genes (Table S3). The enhanced expression of K^+^ transporter genes could contribute to increased accumulation of K^+^ and a higher K^+^/Na^+^ ratio in OE‐miR319 plants. K^+^ could also serve as an important component of osmotic adjustment when plants suffer from salt stress, which balances the K^+^/Na^+^ ratio correlating with plant salt tolerance (Jiang *et al*., 2013; Zhu, 2002). *HAK5* was induced by ethylene to maintain K^+^/Na^+^ homeostasis in *A. thaliana* and positively regulated salt tolerance (Jiang *et al*., 2013). In this study, the *HAK5* homologous gene *PvHAK5* was up‐regulated by salt stress and ET treatment and highly expressed in OE‐miR319 plants compared to WT controls, which may be advantageous for OE‐miR319 plants to absorb more K^+^ for enhanced salt stress tolerance.

In addition to ethylene‐dependent salt tolerance mechanisms, the expression levels of some ethylene‐independent salt tolerance‐related genes were also changed in OE‐miR319 plants, such as ion transporter‐encoding genes (e.g. *NHX*,* VCX1*) and transcription factors (e.g. NFYC, HSP20, MADS, MYB) known to be associated with plant salt tolerance (Chen *et al*., 2015; Guo *et al*., 2016; He *et al*., 2012; Huang *et al*., 2017; Sato and Yokoya, 2008; Xiao *et al*., 2003). It was previously reported that miR319 enhanced plant salt tolerance by altering plant morphology, such as increasing leaf wax and altering root length, in creeping bentgrass (Zhou *et al*., 2013). Wider leaf blades and shorter roots were also observed in OE‐miR319 and 5sr switchgrass. Interestingly, besides enhanced salt tolerance, the miR319‐*PvPCF5* module also enhanced switchgrass above‐ground biomass under normal or salt stress conditions, an additional benefit to a biofuel plant.

**Figure 10:**
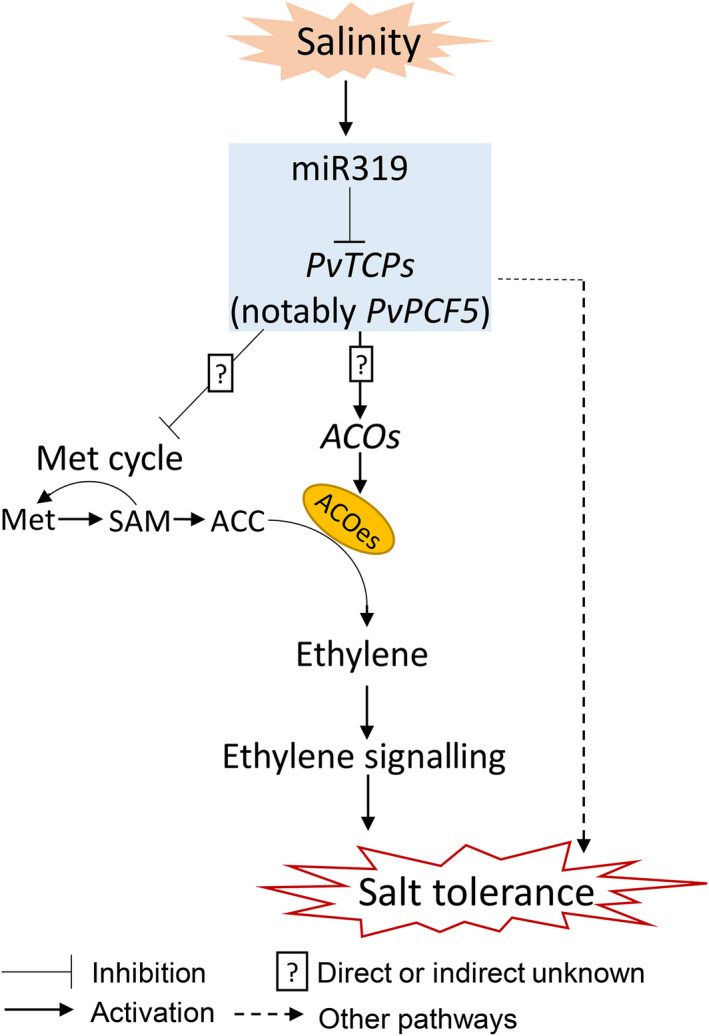
A proposed model of the miR319‐*PvPCF5* module enhancing the salt tolerance of switchgrass by mediating ET biosynthesis and signalling pathways. MiR319‐*PvPCF*s repress the expression level of Met cycle‐related genes and upregulate the expression level of *ACO* genes. Ethylene signaling‐independent pathways also exist in miR319‐*PvPCF*5 module‐mediated salt tolerance.

Overall, our results show that the miR319‐*PvPCF5* module regulates salt tolerance in switchgrass by enhancing ET synthesis and its signalling transduction (Figure 10). OE‐miR319 or repression of *PvPCF5* promotes the expression of salt tolerance‐related genes such as genes encoding PODs and HAK transporters. The miR319‐*PvPCF5* module also suppresses the expression of Met cycle‐related genes, which could be a mechanism of avoiding the overproduction of ET and fine‐tuning ET signalling.

## Materials and methods

### Plant materials and growth conditions

The switchgrass cultivar Alamo was used in this study. Following the *Agrobacterium*‐mediated transformation procedure established in our laboratory (Liu *et al*., 2015), transgenic plants generated from the same callus line as the WT plants were cultured in a greenhouse (natural light and temperature ranging from 25 to 35 °C at night and during the day). In the greenhouse, T1 seeds were harvested by hand‐pollinating the transgenic plants with a WT plant that grew from seed (Liu *et al*., 2017).

### Plasmid construction and plant transformation

For miR319 overexpression, a binary vector containing *Osa‐MIR319b* cDNA (AK241923), as previously reported by Yang *et al*. (2013), was used for transformation (Figure S1a). To block miR319, an artificial target mimicry form against miR319 (*MIM319*) (Data S1) was engineered from the *A. thaliana IPS1* gene (AF236376.1) as previously reported (Franco‐Zorrilla *et al*., 2007; Todesco *et al*., 2010) and cloned into the binary vector pZH01 (Figure S1a). To suppress the function of *PvPCF5a* (Pavir.J03475), a target gene of miR319 in switchgrass, the gene was amplified with the primers PvPCF5(SRDX)_XbaF and PvPCF5(SRDX)_BamR and then linked to a sequence encoding the SRDX domain and cloned into the vector pZH01 for gene expression repression (Hiratsu *et al*., 2003; Xie *et al*., 2017). All of these target genes were under the control of the CaMV 35S promoter (Figures S1a, S6a). The constructs were introduced into the *A. tumefaciens* strain EHA105, separately, for plant transformation.

### Isolation of plant DNA and RNA and molecular tests

Genomic DNA was extracted from leaves of switchgrass plants using the CTAB method for PCR tests as previously reported (Liu *et al*., 2015). Total RNA was isolated from young leaves using TRIzol reagent. One microgram of RNA was used for the synthesis of first strand cDNA for gene expression analysis (Takara RR047A). For analyses of mature miR319 by quantitative stem‐loop RT‐PCR, the miR319 stem‐loop RT‐PCR primer was used for the synthesis of first strand cDNA by a reverse transcription reaction. A nuclear small RNA U6 cDNA (Pavir.J34795.1) was used for RNA normalization for miR319 quantitative stem‐loop RT‐PCR testing (Liu *et al*., 2017; Varkonyi‐Gasic and Hellens, 2011). SYBR green supermix (Takara RR420) was used for quantitative real‐time (qRT) PCR analysis. The reaction was performed using the EcoTM Real‐Time PCR System (Illumina, EC‐100‐1001, CA), as we previously reported (Liu *et al*., 2017). The primers used in the experiments are listed in Table S1.

### Salt tolerance assay

We first tested the salt tolerance of WT and two OE‐miR319 lines TG20 and TG21, in a sand‐culture system. The plants were propagated by splitting tillers. After trimming shoots and roots to approximately 5 cm, tillers were planted in pots filled with pure silica sand. The plants were grown in a greenhouse for approximately 4 weeks until most of newly generated shoots had grown to the E2 stage (Moore *et al*., 1991). Then, the plants were watered with 250 mL of 1/4 Hoagland's nutrient solution supplied with 300 mm NaCl every other day (Guan *et al*., 2018; Huang *et al*., 2017). During salt stress treatment, plant leaves were sampled at 0 h, 0.5 h, 3 h and 6 h for RNA extraction and real‐time PCR testing of the expression of miR319 and its target genes. When exposed to salt stress treatment for 30 days, the shoots and roots of plants were harvested, separately, for K^+^ and Na^+^ content measurement using a flame photometer (410) (Hardin *et al*., 2013; Zhang and Wang, 2015).

In a water‐culture system, we compared the salt tolerance of WT plants, an OE‐miR319 line (TG21) and three *Mim319* transgenic lines (M1, M3, M4), and we also compared the salt tolerance of WT and *PvPCF5‐SRDX* transgenic (5sr) plants. Plants at the E2 stage were cultured in 1/4 Hoagland's nutrient solution and were treated with additional 0 or 300 mm NaCl for fifteen days. The leaves and roots were collected for K^+^ and Na^+^ content measurement with a flame spectrophotometer (410) (Wang and Zhao, 1995). The root tips were stained with diaminobenzidine (DAB) solution to analyse and test H_2_O_2_ accumulation quantitatively (Cen *et al*., 2016; Liu *et al*., 2017).

### Ethylene production assay

The detached first mature leaves of WT and transgenic switchgrass plants (E3 stage) were used to measure ethylene production (Moore *et al*., 1991). To avoid wound‐induced ethylene production, the detached leaves were soaked in double‐distilled water for 24 h before analysis, and then, each leaf (approximately 0.5 g) was incubated in a 14‐mL airtight bottle containing 1 mL of double‐distilled H_2_O or 300 mm NaCl for 3 h, 6 h, 12 h or 14 h in the dark at 25 °C. Then, 2 mL of head space gas was withdrawn using a gas‐tight hypodermic syringe for ethylene production measurement by a gas chromatograph (GC17A, Shimadzu, Kyoto, Japan; Xue *et al*., 2008). Three biological replicates and six technical repeats were performed.

### Transcriptome analyses

To explore the potential molecular mechanisms of miR319‐mediated improved salt tolerance in switchgrass, RNA sequencing of WT (WT1 and WT2) and OE‐miR319 lines (TG20 and TG21) was performed with two biological replicates. Leaves of the second internode from three E2 stage tillers were collected and used for total RNA extraction. Four RNA samples were sent to Beijing Novogene Bioinformatics Technology Co. for sequencing with the Illumina Hiseq 2500 platform. Clean data were obtained from raw data after removing adapters or poly‐N and other low‐quality reads. Under the criteria of an adjusted *P*‐value < 0.05, differentially expressed genes (DEGs) between WT and OE‐miR319 plants were analysed as previously reported (Wang *et al*., 2017). Ten genes were selected to verify their expression levels in the transcriptome by qRT‐PCR using the primers listed in Table S1. The relative expression levels of genes were determined using the 2^−∆∆CT^ method and used for heat‐map production (Livak and Schmittgen, 2001). A ubiquitin gene (AP13CTG25905) of switchgrass was used as the internal control for RNA normalization for gene expression testing (Wuddineh *et al*., 2015).

### ET and ACC treatments

We used WT seedlings as plant materials to test the responses of miR319 and its target genes to exogenous ET or ACC treatment. WT and T_1_ seeds were germinated on two layers of filter paper soaked with (for T_1_ seeds) or without 100 mg/L hygromycin B in a growth chamber at 37 °C for 2 days. The germinated seeds were then transferred onto wet filters at 25 °C for further growth or subjected to combined treatments to analyse the effects of ethylene on the salt tolerance of WT and T_1_ seedlings (Liu *et al*., 2017). A pool of ten or twenty seedlings (sample size *n* = 10 or 20) was treated as one biological replicate for gene expression analysis or the measurement physiological parameters, respectively. Three biological replicates were included in the experiment.

To explore the responses of miR319 and its target genes to ethylene treatment, fifteen 7‐day‐old WT seedlings were selected and placed into airtight bottles with two‐layer wet filter paper and 10 ppm of ethylene gas injected by a hypodermic syringe and incubated at 25 °C (light/dark, 16 h/8 h) for 0, 1, 3, 6, 12 and 24 h. The expression of miR319 and its target genes was examined.

To test the effects of ethylene on switchgrass salt tolerance, sterilized WT seeds were germinated in a growth chamber at 37 °C for 2 days and then treated with 150 mm NaCl combined with different concentrations of ethylene gas (0, 0.5, 1.0, 2.0, 4.0, 8.0, 10 μL/L) or ACC (0, 10, 20, 40, 80 and 100 μm) for seven or fifteen days. Shoot and root lengths, the accumulation of H_2_O_2_ (Liu *et al*., 2017), and the expression of five genes in ET signalling (*PvEIL1, PvEIN3, PvERF1, PvHAK5 and PvPOD53*) and two salt response genes (*PvSTH2, PvNHX*) were measured 7 day after treatment.

To compare the salt tolerance and the effects of ethylene on WT and transgenic switchgrass T_1_ seedlings, 7‐day‐old seedlings were selected and further treated with 300 mM NaCl combined with different concentrations of ACC (0, 20, 100 μm) or 10 μm AgNO_3_ solution for 7 days. Thirty seedlings were placed on two layers of wet filter paper in one 250‐mL bottle. Three seedlings from each bottle were then randomly selected and stained with diaminobenzidine (DAB) solution to observe H_2_O_2_ accumulation in the tissues. H_2_O_2_ and Na^+^ contents were also measured (Cen *et al*., 2016; Liu *et al*., 2017).

### Sequence and phylogenetic analysis

The full‐length amino acid sequences used in this study were obtained from Phytozome ( www.phytozome.net) or NCBI ( www.ncbi.nlm.nih.gov; Table S2). These sequences were used to create an alignment with Clustal W. The phylogenetic tree of TCP/PCF was generated by the MEGA 5.0 program. The amino acid sequences were analysed by the DNAMAN multiple sequence alignment process.

### MiR319 cleavage site analysis

A 5′ RLM‐RACE was used to detect the miR319 cleavage site in miR319 target genes and was performed according to the reported protocol (Wang and Fang, 2015). The gene‐specific primers (GSPs) and 5′ adaptor sequences are listed in Table S1. The target DNA fragment was purified and cloned into the pMD 19‐T vector (Takara). At least 18 positive clones of each gene were picked for sequencing.

### Statistical analysis

In the experiments, data were collected from three biological replicates (*N* = 3) with three times of repeated measurements (*n* = 3), while in the ethylene production assay six times of repeated measurements (*n* = 6) was used for statistical analysis by one‐way ANOVA. Treatments were compared by Duncan's multiple range test (*P *<* *0.05). PROC GLM for ANOVA in SAS 8.2 (SAS Institute, Cary, NC) was used for analyses.

## Author contributions

Y.L. and W.Z. conceived and designed the experiments; Y.L., J.Y. and W.X. performed the experiments; Y.L., D.L. and H.L. analysed the data; Y.L. and W.Z. wrote and revised the manuscript; and all authors read and approved the final version of the manuscript.

## Conflict of interests

The authors declare that they have no conflict of interests.

## Supporting information


**Figure S1** Generation and molecular tests of switchgrass transgenic plants.
**Figure S2** MiR319 positively regulates leaf blade width and biomass yield.
**Figure S3** Salt tolerance tests of switchgrass plants in silica‐ and liquid‐culture conditions.
**Figure S4** Dose‐dependent effects of additional ethylene on salt tolerance of the WT switchgrass seedlings.
**Figure S5** Phylogenetic analysis of TCP (/PCF) proteins of switchgrass, rice and *Arabidopsis thaliana*.
**Figure S6** Generation of the *PvPCF5* suppressed transgenic plants.
**Figure S7** Repression of *PvPCF5* (5sr) enhanced leaf blade width and biomass yield in switchgrass.
**Figure S8** Na^+^ and K^+^ concentration of the *PvPCF5‐SRDX* transgenic plants (5sr) and WT before and after salt treatments for 15 day.
**Figure S9** Identification of the DEGs between WT and OE‐miR319 plants (TGs).
**Figure S10** Transcriptome analysis of WT and OE‐miR319 plants (TGs) using RNA sequence.
**Table S1** The primers used in the experiments.
**Table S2** List of the accession numbers of seven putative salt response genes in ET synthesis and signalling in switchgrass, *Arabidopsis* and rice.
**Table S3** The selected DEGs related to ethylene biosynthesis, signalling transduction and stress resistance.
**Data S1** The sequence of artificial target mimicry microRNA319 (*MIM319*) and sequences alignment with miR319.
