# Supplementary material for: MiR319 mediated salt tolerance by ethylene
Source: Plant Biotechnol J. 2019 Jun 7;17(12):2370–83. doi: 10.1111/pbi.13154 (PMC6835123; doi:10.1111/pbi.13154)
Supplement: Supplementary file 1 — Figure S1 Generation and molecular tests of switchgrass transgenic plants. Figure S2 MiR319 positively regulates leaf blade width and biomass yield. Figure S3 Salt tolerance tests of switchgrass plants in silica‐ and liquid‐culture conditions. Figure S4 Dose‐dependent effects of additional ethylene on salt tolerance of the WT switchgrass seedlings. Figure S5 Phylogenetic analysis of TCP (/PCF) proteins of switchgrass, rice and Arabidopsis thaliana. Figure S6 Generation of the PvPCF5 suppressed transgenic plants. Figure S7 Repression of PvPCF5 (5sr) enhanced leaf blade width and biomass yield in switchgrass. Figure S8 Na+ and K+ concentration of the PvPCF5‐SRDX transgenic plants (5sr) and WT before and after salt treatments for 15 day. Figure S9 Identification of the DEGs between WT and OE‐miR319 plants (TGs). Figure S10 Transcriptome analysis of WT and OE‐miR319 plants (TGs) using RNA sequence. Table S1 The primers used in the experiments. Table S2 List of the accession numbers of seven putative salt response genes in ET synthesis and signalling in switchgrass, Arabidopsis and rice. Table S3 The selected DEGs related to ethylene biosynthesis, signalling transduction and stress resistance. Data S1 The sequence of artificial target mimicry microRNA319 (MIM319) and sequences alignment with miR319. [file PBI-17-2370-s001.docx]

**Supplementary data**

Supplementary data 1 The sequence of artificial target mimicry microRNA319 (*MIM319*) and sequences alignment with miR319.

1 AAAACACCAC AAAAACAAAA GAAAAATGGC CATCCCCTAG CTAGGTGAAG AAGAATGAAA

61 ACCTCTAATT TATCTAGAGG TTATTCATCT TTTAGGGGAT GGCCTAAATA CAAAATGAAA

121 ACTCTCTAAT TAAGTGGTTT TGTGTTCATG TAAGGAAAGC GTTTTAAGAT ATGGAGCAAT

181 GAAGACTGCA GAAGGCTGAT TCAGACTGCG AGTTTTGTTT ATCTCCCTCT AGAAAGGGAG

241 CTCC**T**CTATT CAGTCCAAAG CTTCGGTTCC CCTCGGAATC AGCAGATTAT GTATCTTTAA

301 TTTTGTAATA CTCTCTCTCT TCTCTATGCT TTGTTTTTCT TCATTATGTT TGGGTTGTAC

361 CCACTCCCGC GCGTTGTGTG TTCTTTGTGT GAGGAATAAA AAAATATTCG GATTTGAGAA

421 CTAAAACTAG AGTAGTTTTA TTGATATTCT TGTTTTTCAT TTAGTATCTA ATAAGTTTGG

481 AGAATAGTCA GACCAGTGCA TGTAAATTTG CTTCCGATTC TCTTTATAGT GAATTCCTCT

541 T

Note: The 236 bp to 258 bp sequence of the Arabidopsis *IPS1* gene (AF236376.1) was replaced with a sequence similar to miR319 sequence (*MIM319*) (underlined).


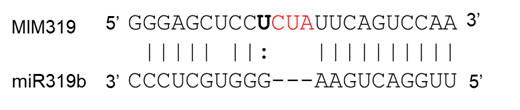


Alignment of the sequence of MIM319 and targeted miR319.

MIM319 are highly complementary to miR319 but contain a 3 nt mismatch loop opposite nts 10-11. Mismatches between MIM319 and miR319 are indicated in red. Nts 10 of the miRNA (site that demarcates cleavage on the target transcript) is highlighted in bold.

**Table S1** The primers used in the experiments.

| **Primer name** | **Primer sequence** |
| --- | --- |
| **Gene clone** | |
| Pre-Y19b_XbaIF | TCTAGAGATGATGTCTTCTCTTCTCTATCC |
| Pre-Y19b_ SalIR | GTCGACGAGAAACAGTAATCACACCAGTG |
| PvPCF5 (SDRX)_XbaF | TCTAGAATGGGCGACGCCGGCCAGT |
| PvPCF5 (SDRX)_BamR | GGATCCGTGGTGAGAAGCCGAGGACA |
| **PCR/RT-PCR** | |
| Osa-MIR319b_F | TGCTGCCGTTTTTCATGTTG |
| Osa-MIR319b_R | GCGTTTCTTGCTTGGCATGT |
| MIM319_F | AAGAAAAATGGCCATCCCCTAGC |
| MIM319_R | GAGGAATTCACTATAAAGAGAATCG |
| 35sF | CGCACAATCCCACTATCCTTC |
| hpt-R | TACTTCTACACAGCCATCGGTCCAG |
| **mature miR319** | |
| Stem-loop RT primer | GTCGTATCCAGTGCAGGGTCCGAGGTATTCGCACTGGATACGACGGGAGC |
| Stem-loop Forward primer | CGGCGGTTGGACTGAAGGGT |
| Stem-loop Reverse primer | GTGCAGGGTCCGAGGT |
| **RNA adaptor and Primers for 5'RLM RACE** | |
| RNA 5’ adaptor | CGACUGGAGCACAGGACACUGACAUGGACUGAAGGAGUAGAAA |
| Oligo(dT) _30_ primer | ATTCTAGAGGCCGAGGCGGCCGACATG-d(T) 30 |
| GeneRacer 5′ primer | AGGACACTGACATGGACTGAAGGAGTAG |
| Universal primers | ATTCTAGAGGCCGAGGCGGCCGACATG |
| PvPCF5-GSP1 | CCTGGTGGGGATGCGGAAAC |

**Table S1** continued.

| **RNA-seq Sequencing ID** | **Forward primer** | **Reverse primer** |
| --- | --- | --- |
| **RNA-seq validation** | | |
| c115588_g1 | CCCACACCCCTTCCGATTT | GGGATGAGGCTGGACTACAACT |
| c55387_g5 | TTTCTCTTGGCTGCCGATG | TCCCAACTTGCTCTGTTTCACT |
| c47827_g2 | CTCCGCAAGCACACCTACCT | CGTCACTGTCTTGTCGAAGAGG |
| c52782_g4 | CGCCGCACATTATGGTCAAG | CCAAAGCCACGCTACGCAAC |
| c47095_g1 | AAAAGGGCGGAGCTTGTAGG | TCGGGGAGATGACTCTGGAG |
| c48643_g1 | TCTTCTACCTTCTCCAGCCCTC | TCAGTTGTCTCCATTCTTTGTGTT |
| c27663_g1 | CTCGCACACCAATCAACAAAA | CACCCCAGGCTCTGAACAAT |
| c53445_g1 | CATAAGAAACTAATGCGGTGGG | TAGGTTCAATCCAATGATAAGCAC |
| c20097_g1 | AGTGCCAGCAGCCTTTGTC | AGCAACGGTGTGGGAGTGGA |
| C35899_g1 | CTGTGATGATGCCAACTGCC | TCCTCCTTGTTCATTGACGCT |
| **RNA-seq ET biosynthesis related genes** | | |
| c58236_g1 | GCTTTCTCGACCATCCATGT | AGCACCTCAGCACCCTCTTA |
| c57698_g1 | CGCCCAAATCTGTTCTTCAT | CTTCTCCTCCATGGCATCAT |
| c51864_g1 | GACTCACCCACCAATCACAA | TATCACCGCTTCACATTGGA |
| c58595_g4 | CACCGGTCAACTTCCTGACT | AGCAAAGGTCGGAAGAACAA |
| c55421_g4 | TTATTGGCACTCGAGGAACC | TCCACACTTTATGGCACAGC |
| c62655_g1 | CAAACTGGCATCGTGCTTTA | CAGCACACTGAACCTGTCGT |
| c56532_g2 | ATCTAGGATCGCAGCAGGAA | ATGGTTGCTGGCATCTGAAT |
| c51639_g1 | TATACGATCAGCGCCAACAA | GCTTACAGCCTTCGAGTTGG |
| c41571_g1 | CTTGGAAGCAGAGGATTGGA | CCGATTTCAGGTCAGCCTAA |
| c49015_g1 | CCTTTGATGGACGACGAGAT | AGGCCGAGAGGAGAGTTGTT |
| c48546_g3 | CTCTCCCAGTCCAGGTTCTC | GGCTGACCAAGGACCACTAC |
| c54468_g1 | GGTACGGGCTTGTAATTTCG | CTAGTCATGTGCCGTGCAGT |

**Table S1** continued.

| **Primer name** | **Forward primer** | **Reverse primer** |
| --- | --- | --- |
| **Primers for ET related genes qRT-PCR** | | |
| PvEIN3 | CAGCAGCAGCAGCAGCAGAG | CAGACCTGAACTGAACCTCAACTCC |
| PvEIL1 | CAGCAGCAGCAGCAGCAGAC | TCCATCATCTTGAGCATGTACTTGAGG |
| PvERF1 | TACGACCGCGCCGCCTAC | GACGAGGAGGACGACGATGAGG |
| PvPOD53 | TGCGAGGACTACTACGACGACAC | TGGAAGTGGAGGCGGATGAGG |
| PvHAK5 | GTCACCACGCTGCTGCTCAC | GCGGCGAAGACGACGAAGAAG |
| PvSTH2 | AGGAGGAGGAGGAGGAGGAGAAC | TCTTGGTCAGGTACTCGGAGATGC |
| PvNHX | GAGAGGTGCCGTGTCCATTGC | ACGACAGTGATTGTGCTGGTGATC |
| **Primers for *PvPCFs* qRT-PCR** | | |
| PvPCF5 | CCATCCAGTTCTACGACGTG | AGCCAGTCGACGGCCTTGCT |
| PvPCF7 | GGCAGCGTCAAGTCCTTGTTCC | TGGTGCTGGTTGGACTGGAGAG |
| PvPCF6 | GCCGAGCACAAGCAGTCCTG | CGACGTGAGAGGTGAAGTTGAAGG |
| PvPCF8 | AGCACCTCCGAGACCAGCAAG | CGAATGAGCCGCCGTTGTAGG |
| PvTCP21 | TGGGTGTCAACAGTAGGGGG | GGGCTGAACAGGAAGGGTATG |
| **Primers of genes used as internal control** | | |
| Ubq | CAGCGAGGGCTCAATAATTCCA | TCTGGCGGACTACAATATCCA |
| U6 | CAGCGAGGGCTCAATAATTCCA | TCTGGCGGACTACAATATCCA |

**Table S2** List of the accession numbers of seven putative salt response genes in ET synthesis and signaling in switchgrass, *Arabidopsis* and rice.

| Gene name | Gene ID | Homologous gene in *Arabidopsis* | Homologous gene in Rice | Reference |
| --- | --- | --- | --- | --- |
| *PvEIL1* | Pavir.Bb03705 | AT2G27050 | LOC_Os07g48630 | Lv *et al.*, 2018; Yang *et al.*, 2015 |
| *PvEIN3* | Pavir.J01001 | AT3G20770 | LOC_Os07g48630 | Lv *et al.*, 2018; Yang *et al.*, 2015 |
| *PvERF1* | Pavir.Ab02556 | AT4G17500 | LOC_Os02g43790 | Shoji *et al.*, 2013; Zhou et al., 2016 |
| *PvHAK5* | Pavir.Bb00115 | AT4G13420 | LOC_Os03g37830 | Jung *et al.*, 2009 |
| *PvPOD53* | Pavir.Ib02646 | AT5G06720 | LOC_Os10g02040 | Zhou *et al.*, 2016 |
| *PvSTH2* | Pavir.Ga01157 | AT1G75540 | LOC_Os04g45690 | Datta *et al.*, 2007 |
| *PvNHX* | Pavir.Ba00176 | AT3G05030 | LOC_Os07g47100 | Bassil *et al.*, 2011; Amin *et al.*, 2016 |

**Reference:**

Amin, U.S.M., Biswas, S., Elias, S.M., Razzaque, S., Haque, T., Malo, R. and Seraj, Z.I. (2016) Enhanced salt tolerance conferred by the complete 2.3 kb cDNA of the rice vacuolar Na^+^/H^+^ antiporter gene compared to 1.9 kb coding region with 5 ' UTR in transgenic lines of rice. *Front Plant Sci.* 7.

Bassil, E., Tajima, H., Liang, Y., Ohto, M., Ushijima, K., Nakano, R. and Esumi, T. (2011) The Arabidopsis Na^+^/H^+^ antiporters NHX1 and NHX2 control vacuolar pH and K^+^ homeostasis to regulate growth, flower development, and reproduction. *Plant Cell* 23，4526.

Datta, S., Hettiarachchi, C., Johansson, H. and Holm, M. (2007) SALT TOLERANCE HOMOLOG2, a B-Box protein in Arabidopsis that activates transcription and positively regulates light-mediated development. *Plant Cell* 19，3242-3255.

Jung, J., Shin, R. and Schachtman, D.P. (2009) Ethylene mediates response and tolerance to potassium deprivation in *Arabidopsis.* *Plant Cell* 21，607-621.

Lv, B., Tian, H., Zhang, F., Liu, J., Lu, S., Bai, M. and Li, C. (2018) Brassinosteroids regulate root growth by controlling reactive oxygen species homeostasis and dual effect on ethylene synthesis in Arabidopsis. *Plos Genet* 14, e1007144.

Shoji, T., Mishima, M. and Hashimoto, T. (2013) Divergent DNA-Binding specificities of a group of ETHYLENE RESPONSE FACTOR transcription factors involved in plant defense. *Plant Physiol*. 162，977-990.

Yang, C., Ma, B., He, S., Xiong, Q., Duan, K., Yin, C. and Chen, H. (2015) MAOHUZI6/ETHYLENE INSENSITIVE3-LIKE1 and ETHYLENE INSENSITIVE3-LIKE2 regulate ethylene response of roots and coleoptiles and negatively affect salt tolerance in rice. *Plant Physiol*. 169**,** 148-165.

**Table S3** The selected DEGs related to ethylene biosynthesis, signaling transduction and stress resistance.

| **Gene_id** | **log2FC** | **padj** | | **Description** |  |
| --- | --- | --- | --- | --- | --- |
| **Ethylene biosynthesis** | | | | | |
| c48546_g2 | 0.87 | 1.59E-25 | 1-aminocyclopropane-1-carboxylate oxidase 1 (ACO1) | | |
| c48546_g3 | 1.66 | 1.67E-14 | ACO1 | | |
| c54468_g1 | 0.87 | 1.04E-09 | ACO1 | | |
| c51215_g1 | 0.79 | 1.13E-63 | S-adenosylmethionine synthase 2 (SAM2) | | |
| c46084_g1 | -0.80 | 1.13E-06 | S-adenosylmethionine synthase 3 (METK3) | | |
| c51215_g1 | 0.79 | 5.54E-61 | S-adenosylmethionine synthase 2 (SAM2) | | |
| c46084_g1 | -0.80 | 2.19E-05 | S-adenosylmethionine synthase 3 (METK3) | | |
| c58236_g1 | -0.86 | 2.69E-59 | S-adenosylmethionine decarboxylase proenzyme (SAMDC) | | |
| c56532_g2 | -0.87 | 2.22E-23 | Spermidine synthase 1 (SPDSYN1) | | |
| c58595_g4 | -0.81 | 1.93E-13 | Spermine synthase (SPMS) | | |
| c51639_g1 | -0.80 | 2.84E-08 | Methylthioribose-1-phosphate isomerase | | |
| c41571_g1 | -0.89 | 1.54E-15 | Probable bifunctional methylthioribulose-1-phosphate dehydratase/enolase-phosphatase E1 (DEP1) | | |
| c49015_g1 | -0.82 | 5.15E-13 | 1,2-dihydroxy-3-keto-5-methylthiopentene dioxygenase 2 (ARD2) | | |
| c62655_g1 | -0.72 | 0.000454 | ARD4 | | |
| c49344_g2 | -0.26 | 0.043137 | Nicotianamine aminotransferase A | | |
| c57698_g1 | -0.39 | 5.96E-12 | S-adenosylmethionine decarboxylase proenzyme (SAMDC) | | |
| c55421_g4 | -0.47 | 1.21E-06 | ARD4 | | |
| c51864_g1 | -0.37 | 8.39E-08 | ARD2 | | |
| c55421_g1 | -0.30 | 0.007216 | ARD4 | | |
| **Ethylene signaling transduction** | | |  | | |
| c51778_g1 | 1.08 | 3.43E-22 | ETHYLENE INSENSITIVE 3-like 1 protein (EIL1) | | |
| c60811_g1 | -0.50 | 5.95E-05 | Ethylene receptor (ETR1) | | |
| c33313_g2 | 0.88 | 1.38E-05 | EIN3-binding F-box protein 1 (EBF1) | | |
| c57867_g1 | -0.67 | 5.69E-11 | Ethylene-insensitive protein 2 (EIN2) | | |
| c63807_g2 | -0.51 | 0.000156 | EIN2 | | |

**Table S3** Continued.

| **Gene_id** | **log2FC** | **padj** | **Description** |
| --- | --- | --- | --- |
| **Oxidative stress related genes** | | |  |
| c31507_g2 | 1.20 | 0.018348 | Peroxidase 66 |
| c39283_g3 | 8.58 | 2.93E-49 | Cationic peroxidase SPC4 |
| c39283_g2 | 6.36 | 8.04E-11 | Cationic peroxidase SPC4 |
| c50843_g2 | 5.34 | 0.000326 | Peroxidase 43 |
| c26442_g1 | 4.36 | 4.70E-08 | Peroxidase 53 |
| c17731_g1 | 3.01 | 0.000572 | Peroxidase |
| c37548_g1 | 2.94 | 3.71E-05 | Peroxidase 15 |
| c53728_g7 | 2.65 | 3.58E-16 | Peroxidase 2 |
| c69850_g1 | 2.27 | 0.0307 | Peroxidase 59 |
| c46013_g1 | 2.04 | 1.34E-11 | Peroxidase 21 |
| c50560_g1 | 1.96 | 8.04E-32 | Peroxidase 37 |
| c53728_g8 | 1.88 | 1.07E-13 | Peroxidase 70 |
| c50225_g1 | 1.87 | 1.78E-32 | Peroxidase 39 |
| c48592_g1 | 1.86 | 3.68E-14 | Peroxidase 4 |
| c53728_g3 | 1.85 | 7.99E-22 | Peroxidase 5 |
| c57232_g1 | 1.83 | 6.85E-99 | Peroxidase 56 |
| c49408_g1 | 1.73 | 5.24E-44 | Peroxidase 1 |
| c53728_g4 | 1.68 | 0.000168 | Peroxidase 2 |
| c31507_g1 | 1.66 | 7.16E-07 | Peroxidase |
| c47862_g1 | 1.64 | 2.74E-19 | Peroxidase 3 |
| c41583_g1 | 1.61 | 0.001827 | Peroxidase 21 |
| c40793_g1 | 1.60 | 6.24E-07 | Peroxidase 24 |
| c60091_g1 | 1.50 | 4.51E-36 | Peroxidase 30 |
| c28539_g1 | 1.42 | 0.007469 | Peroxidase 2 |
| c48578_g1 | 1.41 | 5.61E-99 | Peroxidase 2 |
| c52597_g1 | 1.35 | 2.65E-46 | Peroxidase 70 |
| c57555_g4 | 1.15 | 0.00212 | Peroxidase 9 |
| c59813_g3 | 1.08 | 3.03E-06 | Peroxidase 35 |
| c37652_g1 | 1.07 | 0.000137 | peroxidase 17-like |
| c53178_g4 | 1.05 | 4.16E-38 | Peroxidase 2 |
| c28130_g2 | -4.44 | 0.01764 | Peroxidase 1 |

**Table S3** Continued.

| **Gene_id** | **log2FC** | **padj** | **Description** |
| --- | --- | --- | --- |
| **Ion exchange** | |  |  |
| c52940_g1 | -1.98 | 2.76E-06 | Sodium/hydrogen exchanger 6 （NHX6） |
| c49524_g1 | -1.31 | 0.009273 | Alkali metal cation/H+ antiporter Nha1 C terminus |
| c63301_g6 | -1.27 | 0.000143 | Predicted K^+^/H^+^-antiporter |
| c58431_g1 | -1.27 | ###### | vacuolar cation/proton exchanger 1a-like |
| c53079_g1 | -1.07 | 0.000124 | probable cation transporter HKT6-like |
| c47044_g3 | 1.23 | 3.72E-18 | potassium transporter 16-like |
| c36838_g1 | 1.63 | 2.66E-12 | potassium transporter 24-like |
| c43743_g2 | 1.64 | 3.45E-12 | potassium transporter 19-like |
| c61407_g3 | 1.79 | 2.32E-05 | potassium transporter 25-like |
| c44214_g1 | 1.87 | 0.000179 | Ca^2+^/H^+^ antiporter VCX1 |
| c57987_g3 | 2.00 | 3.69E-22 | Probable potassium transporter 13 （HAK13） |
| c115588_g1 | 2.73 | 0.037554 | Ca^2+^/H^+^ antiporter VCX1 |
| **Transcription factor** | |  |  |
| c48643_g1 | -5.75 | ###### | Transcription factor ORG2 |
| c50095_g1 | 1.07 | 3.83E-05 | transcription factor TGA |
| c45668_g1 | 1.14 | 0.000874 | transcription factor TGA |
| c39018_g1 | 1.04 | 1.66E-38 | nuclear transcription factor Y subunit C-4-like (NFYC-4 like) |
| c39018_g1 | 1.04 | 1.66E-38 | NFYC-4 like |
| c47832_g1 | 1.12 | 4.70E-86 | NFYC-1 |
| c47832_g1 | 1.12 | 4.70E-86 | NFYC-1 |
| c53790_g2 | 1.48 | 6.66E-27 | MYB |
| c49795_g1 | 2.20 | 2.47E-22 | MYB |
| c47873_g1 | 3.92 | 1.22E-06 | MYB |
| c54953_g2 | 1.20 | 2.00E-51 | MADS-box transcription factor |
| c50793_g4 | 1.76 | 4.58E-38 | MADS-box transcription factor |
| c53578_g2 | 1.79 | 4.48E-22 | HSP20 family protein |
| c52251_g1 | 1.80 | ###### | HSP20 family protein |
| c4559_g1 | #NAME? | 0.004503 | Ethylene-responsive transcription factor RAP2-3 |
| c52782_g4 | 2.80 | 4.77E-07 | Ethylene-responsive transcription factor ERF071 |
| c59698_g1 | 1.10 | 0.014118 | ERF055 |
| c49361_g2 | 1.63 | 0.00083 | ERF039 |
| c49361_g3 | Inf | 0.020957 | ERF021 |
| c46712_g1 | 1.17 | 3.02E-06 | ERF12 |
| c55387_g5 | -1.09 | 0.000648 | Dehydration-responsive element-binding protein 1A (DREB1A) |
| c59657_g3 | 1.22 | 4.33E-07 | DREB1E |
| c47827_g1 | 1.02 | 0.000344 | AP2/ERF and B3 domain-containing transcription repressor RAV2 |


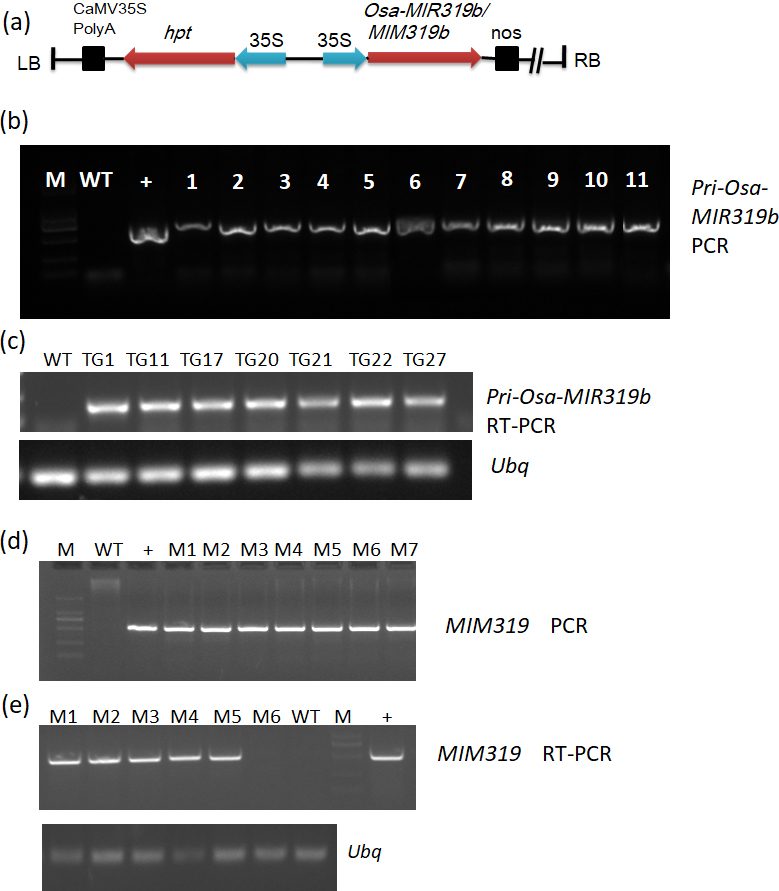


**Figure S1** Generation and molecular tests of switchgrass transgenic plants.

(a) The schematic map of the T-DNA region of the *Osa-MIR319b/ MIM319* expression vector pZH01-*Osa-MIR319b/ -MIM319*; 35S, CaMV 35S promoter; nos, nos terminator; LB, left border; RB, right border; *hpt*, hygromycin phosphotransferase gene. An example of PCR (b) and RT-PCR (c) tests of *Osa-MIR319b* gene in the transgenic plants (TGs). An example of PCR (d) and RT-PCR (e) tests of the target mimicry of miR319 (*MIM319)* transgenic plants (Ms). A ubiquitin gene (*Ubq*) of switchgrass was used as an internal control. WT, wild type plant DNA/ cDNA was used as negative control; +, represented the plasmid DNA used as PCR/ RT-PCR template; M, molecular marker.


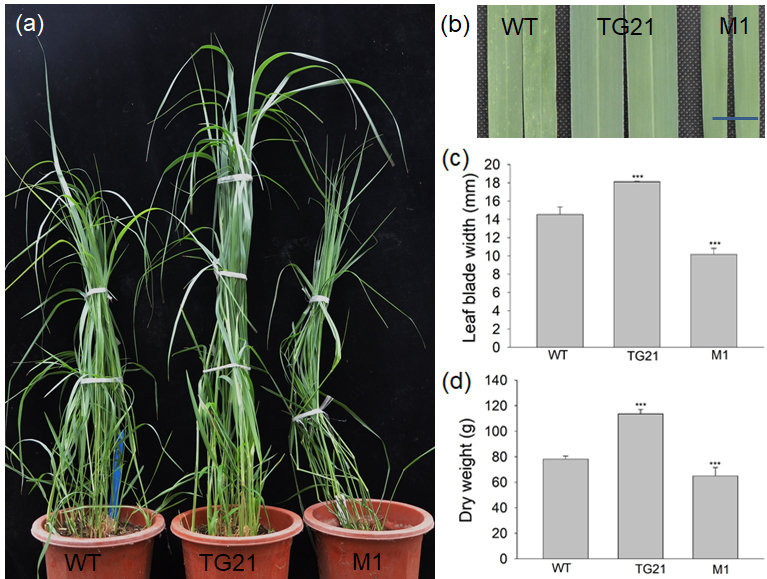


**Figure S2** MiR319 positively regulates leaf blade width and biomass yield.

(a) An example of phenotype comparison of OE-miR319b plant (TG21) and *MIM319* plant (M1). (b) The widest position of the full-developed flag leave of the E3 stage WT, TG21 and M1 plants. Bar = 1cm. (c) Statistical analysis of leaf blade width of the widest position of the full-developed flag leave. Three biological repeats, twenty technical repetitions. (d) Comparison of dry biomass weight of above-ground part of six-month-old switchgrass. The data showed as mean ± SD (N=3, n=3). “***” indicated significant differences by one-way *ANOVA* (*P*<0.001).


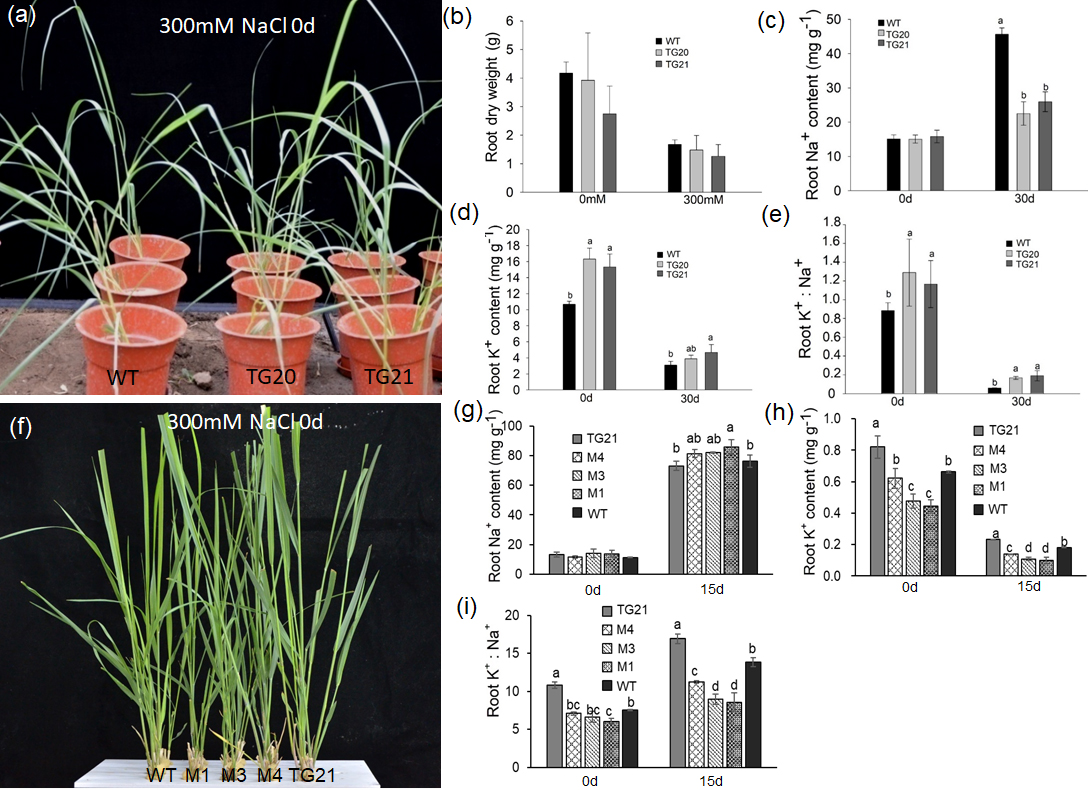


**Figure S3** Salt tolerance tests of switchgrass plants in silica and liquid-culture conditions.

(a) The WT and *Osa-miR319b* transgenic (TG) switchgrass plants that in similar size cultured in pure sand were used for salt tolerance tests. (b), Root dry weight was measured after salt treatment for 30 days. (c, d, e) Comparison of root Na^+^ (c), root K^+^ (d) and the ratio of K^+^/Na^+^ content (e) in WT and TG plants before and after 300 mM salt treatment for 30d. (f) The WT, *MIM319* (Ms) and OE-miR319 (TG21) plants were cultured in 0.5×Hoagland’s nutrient solution before salt treatment. (g, h, i) Root Na^+^ (g), K^+^ (h) and the ratio of K^+^/Na^+^ (i) in the tested plants before and after 300 mM salt treatment for 15 d. The data showed as mean ± SD (N=3, n=3). Different letters indicated significant differences by one-way *ANOVA* and Duncan's multiple comparisons post hoc analysis (*P*<0.05).


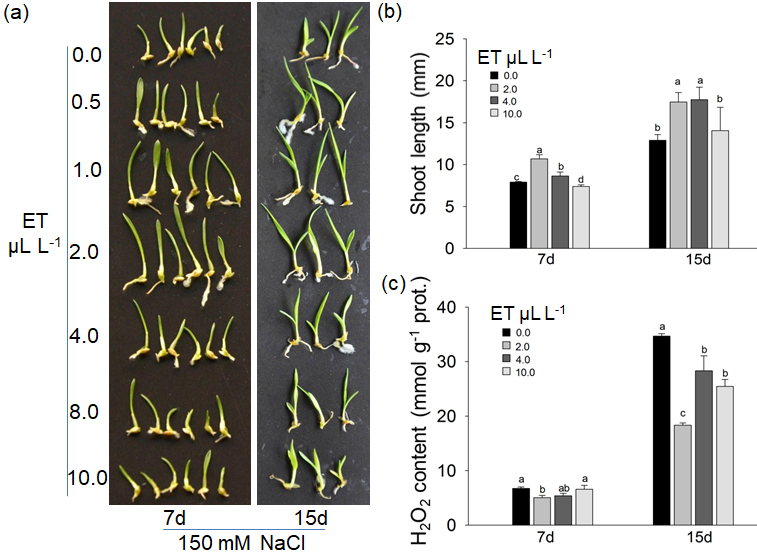


**Figure S4** Dose dependent effects of additional ethylene on salt tolerance of the WT switchgrass seedlings.

1. The phenotype of WT switchgrass seedlings germinated under 150 mM NaCl treatment and combined with additional different concentrations of ethylene for 7 d and 15 d. (b) Comparison of shoot-length of the germinated seedling subjected to the combined treatments with 150 mM NaCl and different concentrations of ethylene for 7 d and 15 d. (c) Comparison of H_2_O_2_ accumulation in the germinated seedling subjected to combined treatments with 150 mM NaCl and different concentrations of ethylene for 7 d and 15 d. The data showed as mean ± SD (N=3, n=20). Different letters indicated significant differences by one-way *ANOVA* and Duncan's multiple comparisons post hoc analysis (*P*<0.05).


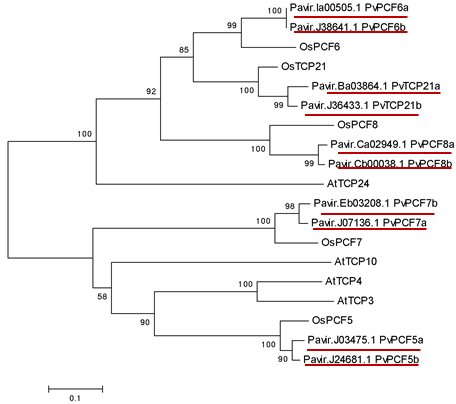


**Figure S5** Phylogenetic analysis of TCP (/PCF) proteins of switchgrass, rice and *Arabidopsis thaliana*.

The protein sequences of PvTCPs, OsTCPs and AtTCPs were used to create alignments with the ClustalW program. MEGA5.0 were used to generate the phylogenetic tree based on the neighbor-joining method. The corresponding gene ID: *OsPCF5* (*Os01g0213800*), *OsPCF6* (*Os03g0785800*), *OsPCF7* (*Os01g0755500*), *OsPCF8* (*Os12g0616400*), *OsTCP21* (*Os07g0152000*), *AtTCP3* (*AT1G53230.1*), *AtTCP24* (*AT1G30210.1*), *AtTCP10* (*AT2G31070.1*), and *AtTCP4* (*AT3G15030.1*).


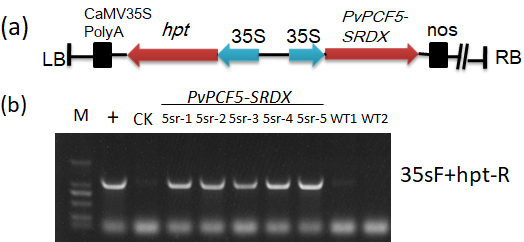


**Figure S6** Generation of the *PvPCF5* suppressed transgenic plants.

(a) The T-DNA region of the plasmids of *PvPCF5* gene repression vectors (*pZH01-PvPCF5-SRDX*). 35S, CaMV 35S promoter; nos, nos terminator; LB, left border; RB, right border; *hpt*, hygromycin phosphotransferase gene. (b) PCR tests of the *PvPCF5*-SRDX transgenic switchgrass plants (5sr). WT, wild type plant DNA was used as negative control; CK, ddH_2_O was used as a blank control; +, represented the plasmid DNA used as PCR template; M, molecular marker.


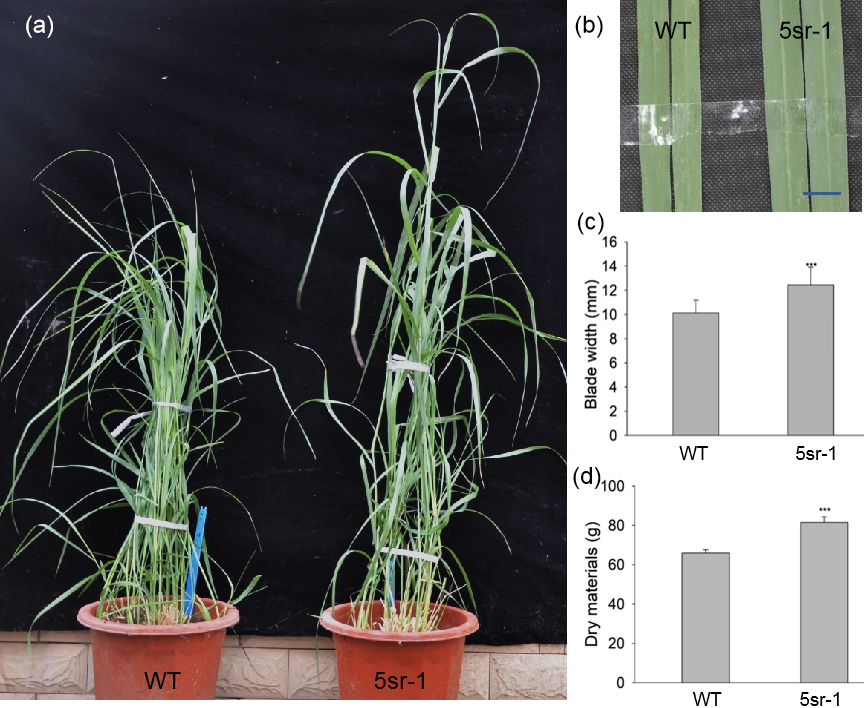


**Figure S7** Repression of *PvPCF5* (5sr) enhanced leaf blade width and biomass yield in switchgrass.

(a) An example of phenotype comparison of WT and repression of *PvPCF5* plant (5sr). (b) Comparison of the full-developed flag leaves from the E3 stage of WT and 5sr-1 plant. Bar = 1cm. (c) Statistical analysis of leaf blade width of the widest position of the full-developed flag leaves. Three biological repeats, twenty technical repetitions. (d) The dry biomass content of above-ground materials of six-month-old switchgrass. Data showed as mean ± SD (N=3, n=3). “***” indicated significant differences by one-way *ANOVA* (*P*<0.001).


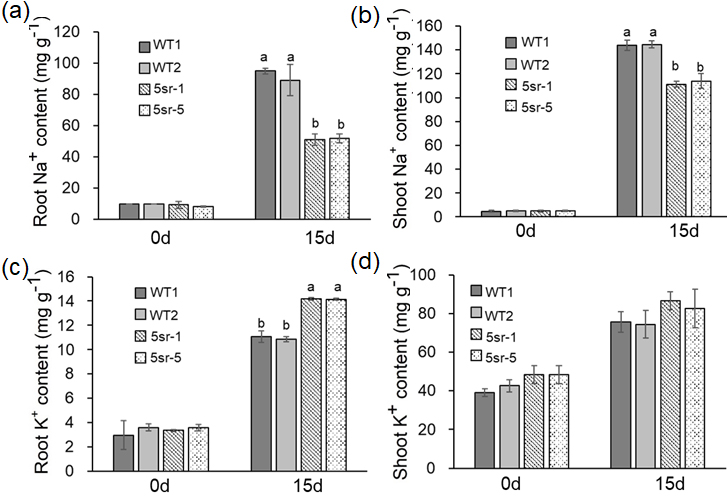


**Figure S8** Na^+^ and K^+^ content of the *PvPCF5-SRDX* transgenic plants (5sr) and WT before and after salt treatments for 15d.

The data showed as mean ± SD (N=3, n=3). The different letters indicated significant differences at the tested time point by one-way *ANOVA* (*P*<0.001).


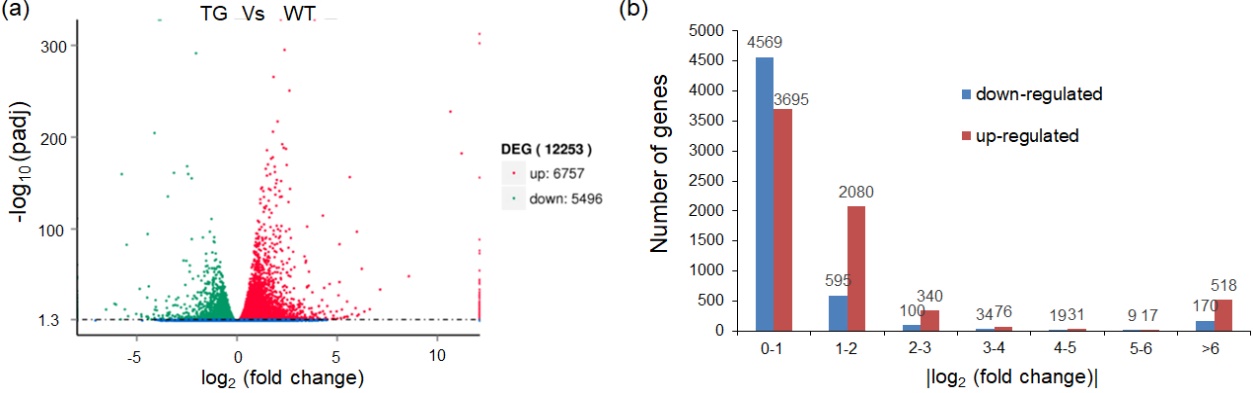


**Figure S9** Identification of the DEGs between WT and OE-miR319 plants (TGs).

(a) Volcano plot of the total DEGs. Green spots indicate down-regulated genes. Red spots represent up-regulated genes. Blue spots indicate genes are not significantly differentially expressed between WT and TG. (b) Statistical analyses of the number of DEGs according to differentially expressed level (log_2_ fold change). The adjusted *P*-value <0.05 as the threshold used to filter DEGs.


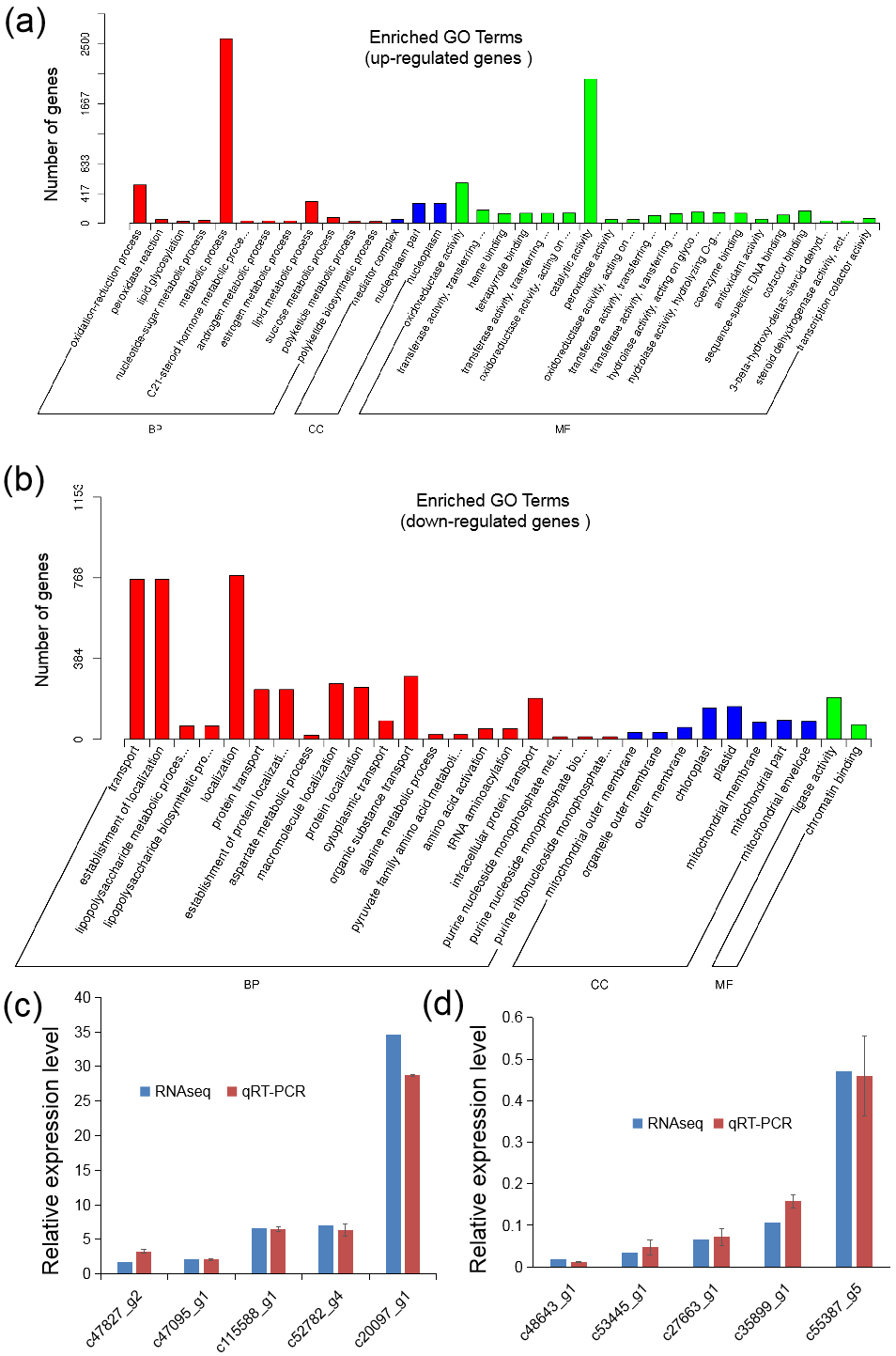


**Figure S10** Transcriptome analysis of WT and OE-miR319 plants (TGs) using RNA sequence.

Functional categories of up-regulated (a) and down-regulated (b) genes in TGs using Gene Ontology (GO) analysis. QRT-PCR validation of random selected five up-regulated genes (c) and five down-regulated genes (d). Two corresponding biological repeats samples used for RNA-seq were performed for qRT-PCR and three technical replicates for each biological repeat were performed. The data showed as mean ± SD (N=2, n=3).
